# Systematic Review of Fatty Acid Composition and the Influence of Coating Media on Fatty Acid Profiles in Canned Fish

**DOI:** 10.3390/md24060204

**Published:** 2026-06-10

**Authors:** Ömer Furkan Kaçar, Okba Hatem, Hüsna Kaya Kaçar, Éva Szabó

**Affiliations:** 1 Doctoral School of Health Sciences, Faculty of Health Sciences, University of Pécs, 7621 Pécs, Hungary; 2 Department of Biochemistry and Medical Chemistry, Medical School, University of Pécs, 7624 Pécs, Hungary; 3 Nutrition and Dietetics Department, Sabuncuoglu Serefeddin Education and Research Hospital, Amasya University, 05100 Amasya, Türkiye; 4 Cochrane Hungary, Medical School, University of Pécs, 7624 Pécs, Hungary; okba.hatem@aok.pte.hu; 5 Nutrition and Food Research Center, Faculty of Medicine, University of Turku, 20014 Turku, Finland; husna.kayakacar@utu.fi; 6 Division of Nutrition and Dietetics, Faculty of Health Sciences, Amasya University, 05000 Amasya, Türkiye

**Keywords:** canned fish, docosahexaenoic acid (DHA), eicosapentaenoic acid (EPA), fatty acid composition, fatty acid exchange, filling medium, lipid migration, nutritional quality

## Abstract

Canned fish products enable long-term preservation of fish, a vital source of eicosapentaenoic acid (EPA) and docosahexaenoic acid (DHA). Despite research on lipid composition, gaps remain in understanding the bidirectional fatty acid (FA) exchange between fish muscle and coating media during processing and storage. After a systematic literature search across five databases (PubMed, Scopus, Web of Science, Wiley Online Library, Cochrane Library), 20 studies were included examining FA profiles across fish species, filling media (vegetable oils, brine, tomato sauce), and storage durations (up to 5 years). Five studies showed that n-3 FAs migrate from fish to the filling medium, enhancing its nutritional value, while fish muscle absorbs FAs from the oil, increasingly resembling the filling medium. The use of n-6 FA-rich oils (sunflower, soybean) lowered the n-3/n-6 ratio in flesh. Conversely, aqueous media (brine) and tomato sauce maintained better ratios. EPA and DHA content generally decreased due to canning and storage, with retention varying by fish species, filling medium, and sterilization method. This review underscores significant FA exchange between fish and filling media, confirming bidirectional lipid interchange during processing. To optimize health benefits, aqueous packing media are recommended to preserve lipid profiles or to consume the covering oil to recover nutrients. Further research is needed on other factors altering FA content in canned fish such as environmental and geographical variables (including catching season), pre-canning preparation and sterilization steps (such as freezing, steaming, and frying), sterilization conditions (time, temperature, F_0_ value) and lipid oxidation induced by thermal processing.

## 1. Introduction

Fatty acids (FAs), whether as components of molecules or functioning independently, play various roles within cells [1]. They are either synthesized through de novo FA synthesis or acquired from external sources [2]. Humans cannot synthesize certain biologically important FAs, specifically omega-3 (n-3) alpha-linolenic acid (ALA) and omega-6 (n-6) linoleic acid (LA), which are therefore classified as essential fatty acids (EFAs). All tissues require FAs for structural integrity and cell membrane formation, with the brain, retina, and other neural tissues being particularly rich in long-chain polyunsaturated fatty acids (LCPUFA) [3].

In recent decades, the importance of these FAs in human nutrition and health has been widely acknowledged [4]. N-3 polyunsaturated fatty acids (PUFAs), particularly eicosapentaenoic acid (EPA) and docosahexaenoic acid (DHA), are crucial dietary nutrients for the prevention of mental, neural, and particularly cardiovascular diseases. As a result, several countries and numerous international and national organizations, including the World Health Organization (WHO), and The American Heart Association (AHA) [5,6] have recommended the inclusion of EPA and DHA in the human diet.

Only a limited number of microalgal species can effectively synthesize the crucial n-3 LCPUFAs, EPA and DHA, which are subsequently consumed by aquatic invertebrates and fish. Consequently, aquatic foods are widely recognized as the primary source of LCPUFA-rich products for humans [7]. This has led to a surge in interest in fish consumption in recent years, owing to the extensive health benefits associated with their high PUFA content [8]. In fact, consumer awareness of the need to increase dietary intake of n-3 LCPUFA has grown significantly over the past decade [9].

Fish has long been valued as an essential part of the diet due to its rich n-3 LCPUFA content [10]. Canned fatty fish offers the benefit of delayed consumption, serving as a complement to fresh options [11]. Canning is a vital method for preserving fish, as it creates shelf-stable products, thereby expanding access and significantly contributing to human nutrition without requiring cold storage [12]. Notably, canned fish is distinguished by its safety, affordability, and long shelf life [13]. A study by Watters and Edmonds emphasizes that canned oily fish is among the most cost-effective sources for meeting the recommended daily intake of EPA and DHA [14].

It is well established that the lipid content and composition of canned fish are directly linked to their quality [15]. In contrast, the FA composition of fish is influenced by geographical factors and environmental conditions, varying with species, fishing grounds, and catching season [16,17]. An expanding body of literature has explored the FA composition of canned fish [13,15,18,19,20]. Additionally, several researchers have examined the effects of the canning process on FA [21,22,23,24,25,26,27,28].

Canned fish products undergo a series of industrial processes, including pretreatment storage methods, such as chilling and freezing, followed by cooking, sterilization, and subsequent storage [22]. As a result, fish are exposed to various conditions that can lead to nutritional and sensory losses in the final product [29]. Furthermore, it has been observed that changes in food quality during storage arise from alterations in physicochemical and microbiological properties, which reduce their nutritional value, palatability, and safety [27]. Over the past decades, studies have provided significant insights into the changes in total fat content and FA composition of canned fish oil during the intense heat treatment involved in sterilization processing. Additionally, these researchers have documented alterations in the FA composition of canned fish oil during canning and storage [13,18,22,23,24,25,30].

Marine lipids, abundant in unsaturated compounds such as PUFAs and cholesterol, are especially susceptible to oxidation. Consequently, the high temperatures involved in pre-cooking and sterilization can initiate oxidative processes in canned fish [31]. These oxidation products are associated with adverse health effects, including increased oxidative stress, inflammation, and potential contribution to the development of chronic diseases [32]. It has been observed that the liquid medium used in canning not only facilitates heat transfer from the retort to the fish muscle but may also modify the lipid profile of the final product [12,33]. Similarly, some studies suggest that the filling medium can cause varying degrees of dilution, partial extraction of certain components, and different heat transfer into fish muscle [15,26]. In this context, researchers have aimed to evaluate the impact of different liquid mediums, such as oil and brine, on the FA composition, cholesterol oxide content, and degradation level of covering oils in the most commonly marketed canned fish products [11,15,23,26]. Furthermore, the type of coating substance such as soybean oil (SO), olive oil (OO), sunflower oil (SFO), or tomato sauce (TS) can affect the chemical composition of the fish, particularly the FA profile, due to interactions between fish lipids and these coating media [23,34].

The degradation levels of oils used in canned fish and the influence of fish type on these oils’ quality have not been thoroughly explored [11]. While extensive research exists on the lipid composition of canned fish, significant gaps persist in our understanding of the bidirectional exchange of FAs between fish and various coating media during processing and storage. Moreover, evidence regarding the release of PUFA into the filling medium remains inconclusive. Numerous studies have highlighted the migration of n-3 PUFAs, such as EPA and DHA, from fish to the filling media and, conversely, the incorporation of n-6 FA from vegetable oils into fish flesh. However, the overall nutritional implications of this exchange are still unclear. The sterilization process, typically conducted at temperatures between 110–130 °C, is a crucial step in canning that ensures microbiological safety and shelf stability [32,35,36]. However, the high thermal load applied during sterilization presents a considerable risk to the lipid fraction of fish [22,32]. Due to their high degree of unsaturation, LCPUFAs, particularly EPA and DHA, are inherently susceptible to oxidative degradation, and thermal treatment may accelerate lipid oxidation [25,37]. This may also result in significant losses of these nutritionally valuable FAs and adversely affect both the nutritional and sensory qualities of the final product [37,38]. Furthermore, there is limited comparison of how different filling media affect the overall nutritional profiles of canned fish products. Therefore, this systematic review aimed to critically evaluate current findings on the interaction between fish and coating media in canned products. The second objective was to assess how these interactions influence the nutritional quality of canned fish, with a particular focus on FA profiles and the comparative impact of various coating materials.

## 2. Results

### 2.1. Results of the Literature Search

**Figure 1:**
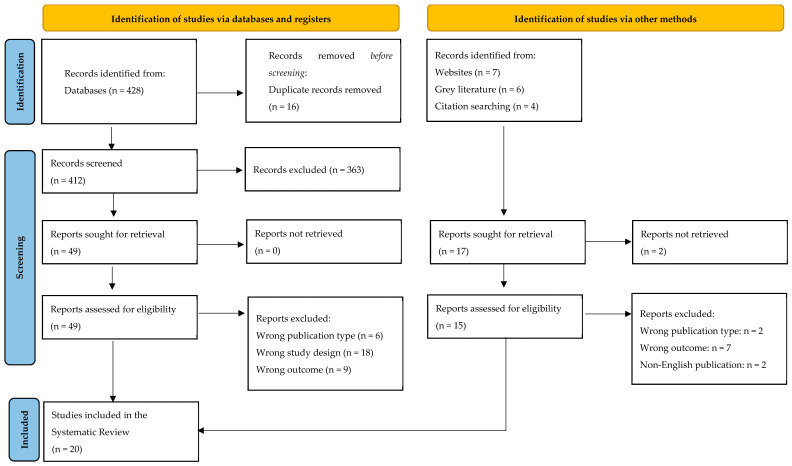
Flow diagram of study selection.

In the scanned databases, 428 unique records were initially identified (Figure 1). After removing 16 duplicates, the remaining 412 records underwent title and abstract screening. 363 records were excluded for not meeting the predefined eligibility criteria, leaving 49 reports to be retrieved. Also, 17 additional records were identified through websites, grey literature, and citation searching; however, 2 of these could not be retrieved. A total of 64 articles (49 from databases and 15 from other methods) underwent full-text evaluation to determine their eligibility for this systematic review. After full-text assessment, 44 articles were excluded. Eight articles were excluded due to incorrect publication types: six were narrative reviews or book chapters [22,27,39,40,41,42], and two were conference abstracts [43,44].

Eighteen studies were excluded due to inappropriate study design. Specifically, six were market surveys or cross-sectional nutritional screenings that reported FA content of commercial canned fish but lacked comparisons between raw and canned samples and did not examine the interaction between fish muscle and the filling medium [23,45,46,47,48,49]. Other excluded studies investigated the effect of natural antioxidants, algae extracts, or other alternative packing additives on the lipid oxidative stability of canned fish, yet they did not measure FA exchange between the fish and the covering liquid [12,50,51,52,53]. Another subgroup focused on the FA characteristics or quality of the filler oil rather than the canned fish muscle itself [11,54,55]. The remaining studies in this category investigated artificial enrichment of canned products with exogenous oil [56], the formation of cholesterol oxidation products [31], a combined market and food safety survey without raw to canned comparison [57], or the application of spectroscopic methods to optimize thermal processing conditions [58].

Sixteen articles were excluded due to wrong outcome. Four studies reported the FA profile of secondary products such as cooking liquid waste or industrially processed tuna and sardine oils [29,59,60,61]. Four further studies investigated lipid oxidation indices or the effectiveness of antioxidant packing media in canned fish, without reporting the FA composition in relation to the interaction with the filling medium [62,63,64,65]. Two studies measured FA as a secondary variable alongside environmental contaminants like mercury and selenium [66,67]. One study assessed the quality of the edible vegetable oil used as the canning medium without reporting changes in the fish muscle FA profile [68]. Three studies reported general nutritional or lipid characterization of canned fish, including cod liver, underutilized species, and sardine meal composition without addressing the relationship between fish and filling medium FA [21,69,70]. One study focused on the impact of prior frozen storage duration on the overall quality of canned fish rather than on the effects of the filling medium [71], and another reported lipid changes during thermal sterilization without comparing raw fish to the canned product in the context of different covering media [72].

Two articles were excluded because they were published in a non-English languages: one in Korean [73] and the other in Spanish [74]. Finally, 20 articles met all the inclusion criteria and were included in the systematic review.

### 2.2. Characteristics of the Studies

**Figure 2:**
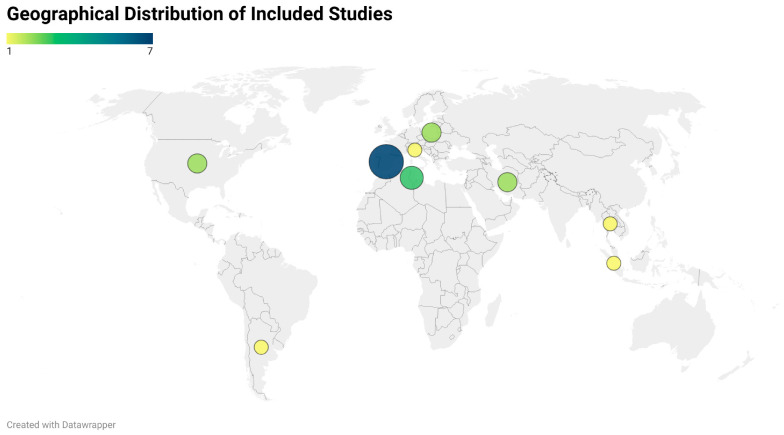
Geographical distribution of the included studies.

The included studies represent a wide range of geographically diverse areas (Figure 2), with a predominance of research conducted in Europe, particularly Spain [15,18,30,33,75,76,77]. Additional European studies were carried out in Italy [34] and Poland [13,37]. African research was represented by studies from Tunisia [25,28,78], while Asian studies included those from Iran [26,79], Thailand [80], and Indonesia [20]. The Americas were represented by studies from the USA [21,24] and Argentina [81].

These studies explored a wide variety of fish species (Table 1), with a particular focus on oily pelagic fish. Tuna species were predominantly studied, specifically albacore tuna (*Thunnus alalunga*) [15,18,24,30], bluefin tuna (*Thunnus thynnus*) [25], and yellowfin tuna (*Thunnus albacares*) [33]. Another primary focus was on small pelagic fish, including sardine (*Sardina pilchardus*) [25,28,33,34,75,78], Spanish sardine (*Sardinella aurita*) [21,78], and anchovy (*Engraulis anchoita*) [81]. Other small pelagics studied included sprat (*Sprattus sprattus* or *Clupeonella cultriventris*) [13,37,79], thread herring (*Opisthonema oglinum*) [21], and herring (*Clupea harengus*) [13]. Additionally, mackerel species were investigated, such as Atlantic mackerel (*Scomber scombrus*) [77] and chub mackerel (*Scomber japonicus*) [21] in some studies. Freshwater or similar species included silver carp (*Hypophthalmichthys molitrix*) [26], catfish (*Pangasius sutchi*) [20], and European eel (*Anguilla anguilla*) [76].

The studies included in the analysis employed various filling media (Table 1) to assess lipid interactions and changes in nutritional quality. Vegetable oils were predominantly used, including SO [15,18,21,26,30,79,81], SFO [13,26,33,37,76,77,79,81], and grapeseed oil (GSO) [28]. OO was frequently examined, often categorized into refined and extra virgin (EVOO) types [25,26,28,33,34,75,76,77,78]. Aqueous media were also utilized, such as brine at varying concentrations [18,20,21,26,33,77,79,80] and water [20,34,77]. Additionally, TS was used as a packing medium in several studies [13,25,78]. One study explored “raw-packed” tuna without any coating material [24], while another used spiced OO containing chili and pepper [76].

Storage durations varied significantly across the studies included (Table 1), ranging from immediate analysis post-processing to multi-year periods. The effects of short-term storage and processing were analyzed at intervals such as 5 days [15], 14 days [13,20], 30 days [30], and 5 weeks [24]. Medium-term storage periods included 2 months [76,81], 3 months [15,18,28,77,78,80], 4 months [26,34], and 6 months [25,75,78,80]. Additionally, long-term storage effects were investigated in some studies, extending to 9 months [80], 11 months [15], 1 year [30,75,76,80,81], and up to 3 to 5 years [30,75,79]. Moreover, some studies did not specify a storage period and focused solely on the immediate effects of sterilization and other processes during canning [21,33,37].

### 2.3. Comparison of Canned and Raw Fish Fatty Acids

All studies included in the analysis investigated changes in FA profiles from raw or initial to canned fish to highlight nutritional alterations (Table 2). The canning process led to significant changes in the FA composition of fish muscle, with variations depending on the species and the type of packing medium used. Regarding saturated fatty acids (SFAs), the trends varied, and in fish preserved in vegetable oils, the percentages often decreased compared to raw samples [13,15,21,25,26,28,30,33,34,37,75,76,77,78,79]. For instance, in Albacore tuna canned in SFO, there was a significant reduction in SFA content [15,30]. A similar decrease was observed in European eels canned in SFO and OO, where the SFA content declined from 32.0% in raw fish to a range of 21.8–27.0% post-canning [76]. In contrast, the level of SFA often remained stable or increased due to the concentration effect caused by moisture loss [20,21,79,80]. In catfish canned in 3% brine, SFA content increased from 0.51% in its raw state to 2.46% after canning [20]. For Spanish sardines canned in brine, the SFA content remained relatively stable, with values of 37.7% and 37.3%, respectively [21].

Regarding monounsaturated fatty acids (MUFA), all studies except one [18] reported significant changes (Table 2). Numerous studies have shown that MUFA levels increase after the canning process, regardless of the species, whether in OO, TS, or aqueous coatings like brine and water [13,20,25,26,28,33,34,75,76,78,80]. For example, in sardines, MUFA levels rose significantly from 20.7% to 50.6% after canning in OO due to the absorption of oleic acid (OA) [75]. Conversely, some studies have noted a decrease in MUFA levels in canned fish preserved in vegetable oil and brine [13,15,21,26,37,77]. In thread herring, MUFA levels dropped from 19.2% to 17.5% after canning in brine [21].

The PUFA component showed the most significant variations (Table 2). In 14 studies examining the packaging of vegetable oils, the total PUFA content in fish increased compared to raw samples [13,15,21,26,28,30,33,37,75,76,77,78,79,81]. For instance, PUFA content increased from 15.6% in raw fillets to 34.9% in samples canned in SFO [26]. However, specific marine n-3 PUFAs (EPA and DHA) generally exhibited a relative decline in percentage when canned in oil (Table 2) [15,18,21,25,28,30,33,34,37,75,76,78,79]. Studies on tuna canned in SO indicated that the total n-3 PUFA content decreased compared to raw samples [15,18,30]. Similarly, the EPA and DHA content in Spanish sardine canned in SO dropped from 4.86% to 0.84% and from 29.46% to 4.34%, respectively [21]. Additionally, EPA and DHA content decreased in sardines canned in OO and TS after the canning process [28,78]. Conversely, the percentages of EPA and DHA were either better preserved or increased in samples canned in brine [21,26,77]. For example, the DHA concentration increased from 17.9% to 20.6% in thread herring preserved in brine [21]. Furthermore, in Atlantic mackerel, DHA levels in brine-canned samples (18.96 g/100 g FA) were higher than in the initial fresh fish (17.03 g/100 g FA) [77].

### 2.4. Interaction Between Fish and Filling Medium Fatty Acids

In our analysis of the FA compositions of both the fish and the filling medium from the included studies, we identified several interactions suggesting the transfer of FAs (Table 3). Ten studies have specifically investigated the mechanism of lipid interchange, confirming that a regrouping of lipids occurs, with FAs migrating bidirectionally between the fish muscle and the filling medium [13,15,26,30,34,37,44,75,76,79,81].

#### 2.4.1. Effect of Fish on Filling Medium Fatty Acid Composition

Regarding the migration from fish to coating material, several investigations demonstrated the enrichment of the filling with marine FAs, such as EPA and DHA (Table 3) [13,15,26,34,37,75,76,79]. To confirm migration from the muscle in Albacore tuna canned in SO, EPA and DHA values, which were absent in the initial oil, were detected in the covering oil after sterilization and storage [15,30]. Similarly, in sardines canned in OO, the filling medium showed increased levels of PUFA and specific marine sterols after the canning process [34]. In canned silver carp, this interaction also resulted in an elevated concentration of EPA and DHA in the filling mediums, such as SO and SFO [26].

#### 2.4.2. Effect of Filling Medium on Fish Fatty Acid Composition

Thirteen studies that compared various filling materials within identical experimental setups emphasized that the coating substance is the primary factor influencing the final nutritional profile [13,18,20,21,25,26,28,33,34,44,76,77,78,79] (Table 2). Additionally, several studies indicated that the canning process leads to the incorporation of FAs from the filling medium into the fish muscle [13,25,26,28,33,76,77,81]. For example, when tuna was canned in SO, the resulting product exhibited a significant reduction in n-3 PUFA content and an increase in n-6 PUFA content due to lipid migration, which includes the transfer of OA and LA from the SO [30]. In silver carp, LA content increased approximately 9-fold, 7.5-fold, and 2.5-fold when canned in SFO, SO, and OO, respectively, with the FA composition of the canned fish tending to mirror the FA profile of the oil used as the filling [26]. Similarly, in Albacore tuna canned in SO, the lipid content was enriched, characterized by increased levels of MUFA and PUFA, following sterilization and storage [30]. Furthermore, sprat packed in SO showed significantly lower SFA and MUFA values but higher total PUFA levels compared to brine-packed samples due to the absorption of LA [79]. The integration of OO into the fish muscle resulted in an increase in MUFA content and a decrease in SFA levels in the lipid composition of canned sardine after prolonged storage [34,75].

The use of OO significantly increased MUFA levels in fish flesh [25,26,28,33,34,75,76,78]. In Atlantic mackerel, packing in EVOO or refined OO led to higher MUFA levels compared to aqueous media, although this increase was not always statistically significant across all fractions [77]. In sardines, canning with OO consistently reduced the SFA fraction while boosting MUFA content [28,34,75].

Findings from several included studies suggest that aqueous or tomato-based filling media generally help maintain a higher nutritional quality or ratio in fish muscle compared to high-n-6 oils [13,21,26,33,77,79] (Table 2). One study demonstrated that silver carp samples canned in brine preserved PUFA and DHA levels better than those in other filling media, such as SO and SFO [26]. In Atlantic mackerel, samples packed in water or brine retained higher levels of EPA and DHA (expressed as g/100 g tissue) compared to those packed in SFO, OO and EVOO [77]. Additionally, in catfish, canning in brine increased the concentrations of SFA, MUFA, and PUFA relative to raw fish due to water loss, without altering the n-3/n-6 balance as drastically as vegetable oils [15,20]. Domiszewski et al. observed that all types of canned fish in TS showed higher values for total n-3 FAs and consequently presented the highest values for the ratio in a study comparing three different fish species canned in SFO versus TS [13]. The lipid content of sardine in TS was found to remain constant after sterilization [78], and TS canned sardine exhibited higher concentrations of EPA and DHA compared to raw samples [25].

In the context of assessing lipid quality, ten studies specifically focused on and calculated the health-relevant ratio of n-3 to n-6 PUFAs [13,26,28,30,37,76,77,79,80,81]. Canning anchovies in the n-6 rich oil (such as SO for canned anchovies and SFO for salted-ripened fillets) led to a significant reduction in the n-3/n-6 ratio, primarily due to the absorption of the covering oil during canning and storage [81]. For silver carp canned for four months, the n-3/n-6 ratio decreased significantly in samples canned in SFO, and moderately in those canned in OO and SO [26]. The n-3/n-6 ratio fell from 1.42 in raw silver carp to 0.24 in samples canned with SFO and 0.42 in those canned with SO [26]. Furthermore, in European eel canned in SFO, the n-6/n-3 ratio increased from 0.41 in raw eels to 10.23 after 12 months of storage [76]. Vegetable oils rich in LA were primarily responsible for the changes in the n-3/n-6 ratio in canned fish [77].

**Figure 3:**
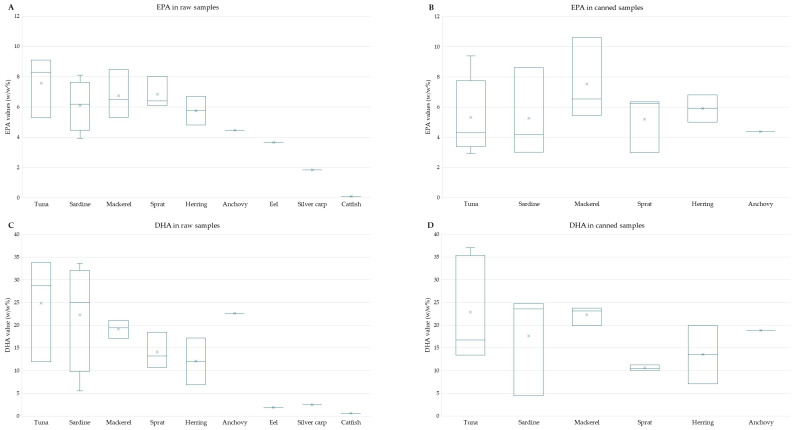
EPA and DHA values in raw ((**A**) and (**C**) respectively) and canned products ((**B**) and (**D**) respectively) by fish species.

The EPA and DHA content in fish vary not only by species but also by the location and timing of the catch. According to the articles included, raw tuna exhibited the highest EPA (9.1 *w*/*w*%) and DHA (33.8%) levels (Figure 3). However, after canning, mackerel showed the highest EPA content (10.61 *w*/*w*%), while tuna retained the highest DHA content (37.08 *w*/*w*%) (Figure 3). It is important to note that these values varied significantly among both raw and canned samples. Freshwater fish had considerably lower EPA and DHA values in both raw and canned forms compared to fatty sea fish (Figure 3). Generally, the range of EPA and DHA values in raw samples was slightly narrower than in canned samples, although the DHA values for tuna and sardines varied widely in both raw and canned forms (Figure 3).

Despite the wide variation in the selected articles regarding fish species, filling medium, and storage duration, most findings pertain to tuna or sardines. Consequently, changes in EPA and DHA values were summarized solely for these two fish species (Table 4). In sardines, both the filling medium (OO, GSO, TS) and storage duration (1 month-5 years) led to a reduction in EPA content across all samples. The most significant percentage change (−72.4%) was reported by Bouriga et al. after 3 months of storage in GSO compared to fresh sardines [28]. The results for DHA content were less consistent: in two studies [28,75], higher DHA levels were observed after canning in OO and subsequent storage compared to the raw samples, whereas other studies noted a decrease in DHA content relative to raw values, with the largest percentage change occurring during thermal sterilization in OO (−57.9%) [33]. For tuna, the findings were more straightforward: both EPA and DHA levels decreased due to the filling medium and/or storage. The most substantial percentage change was observed in tuna canned in SO after 3 years of storage (EPA: −63.9%, DHA: −67.6%) [30].

### 2.5. Effect of Storage Time

Among the included studies, 11 specifically assessed the impact of the storage period [15,25,28,30,34,37,76,77,79,80,81]. In the short to medium term (0–6 months), significant changes in FA profiles (Table 2) were noted, such as an increase in LA content and a decrease in DHA values, within the first month of storage in Albacore tuna canned in SO [30]. Conversely, in sardines canned in OO, a notable alteration in the lipid profile was observed after 6 months, where the FA composition of the sardine flesh shifted to resemble the coating oil, particularly showing a significant increase in MUFA content and a loss of SFA values [75].

A study on long-term storage (1–5 years) found that in tuna canned in brine, total PUFA, n-3 PUFA, and n-6 PUFA concentrations significantly declined in a stepwise manner over 12 months, with DHA levels notably dropping after 6 months [80]. In contrast, sprat canned in SO and stored for 3 years showed a marked decrease in SFA, MUFA, and n-3 PUFA content compared to cooked fish, while n-6 PUFA levels increased due to ongoing interaction with the covering oil [79]. On the other hand, sardines canned in OO for 5 years maintained a stable FA profile after an initial 6-month equilibration period [75].

Beyond the initial canning effect, the duration of storage significantly influences the stability of FAs, particularly the unsaturated types [25,79,80] (Table 2). For instance, in canned sprat, extended storage in both SO and brine resulted in a reduction of PUFA, EPA, and DHA content compared to freshly canned samples [79]. Similarly, tuna canned in brine and stored for 12 months showed a decline in PUFA, EPA, and DHA content, while SFA and MUFA levels increased [80]. In the case of Albacore tuna canned in SO and stored for up to 3 years, there was a rise in the percentage of SFA and MUFA values, along with a decrease in PUFA, EPA, and DHA contents [30].

Pre-canning preparation steps, such as cooking, steaming, or frying, primarily affect the proximate composition (moisture and fat content) but typically result in minimal initial changes to the FA composition percentages [26,30,37,44,79] (Table 2). A study by Selmi et al. found that cooking tuna did not significantly alter the composition of MUFA, PUFA, EPA, or DHA, with statistically significant changes observed only in SFA values [25]. In sprat, both smoking and steaming did not significantly impact the proportion of EPA and DHA in lipids [37,79]. Conversely, steaming white tuna nearly doubled the fat content on a dry matter basis due to water loss; however, the steaming process itself did not significantly change the FA composition compared to the raw material [30]. Additionally, Gómez-Limia L et al. reported that frying European eels led to a “significant increase” in total lipid content, with levels rising from approximately 11.7% to between 17% and 19% [76]. Furthermore, cooking was noted for causing significant fat loss among the included studies [13,25,37,81].

## 3. Discussion

### 3.1. Effects of Filling Medium and Fish Species

In this systematic review, we summarize the current evidence on how canning and storage affect the FA composition of various fish species. An increasing body of literature highlights the significance of FA composition and its alterations during the processing and storage of canned fish. The most notable outcome of the canning process is the bidirectional interchange of lipids and FAs between the solid fish muscle and the liquid filling medium [15]. The migration and regrouping lead to a measurable decrease in these key FA values in the fish flesh, while simultaneously enriching the nutritional value of the covering liquid, especially oily media [15,30]. This phenomenon is often attributed to the effects of heat sterilization on FA stability [21,25,30]. This interchange determines the nutritional quality of the final product [34]. A recent study conducted by Domiszewski Z & Mierzejewska S clarified that EPA and DHA are regrouped from fish into oils during canned fish production [37]. Similarly, it was emphasized that changes in FA composition occur due to migration from fish to coating oil at various stages [30]. Numerous studies have noted the significant influence of the filling medium on the FA profile of canned fish, as demonstrated by the exchange of FAs between the liquid medium and fish muscle [21,23,25,26,31]. These findings offer valuable insights into how using oil as a packing medium can significantly affect the lipid content and FA profile of the final product. In the included studies we also observed that most FA classes were lower in fish samples canned in vegetable oils compared to raw samples.

Moreover, in oil-packed products, the fish muscle notably incorporates exogenous FAs from the covering medium [26,30]. This results in the muscle profile mirroring that of the filling medium [26]. For example, the use of vegetable oils leads to the uptake of OA and LA [30], with studies indicating that LA content increases approximately 9-fold in silver carp canned in SFO [26]. In the present systematic review, we also found that the FA composition of the filling medium influenced the FA composition of the canned fish. For instance, fish canned in SO, which is rich in n-3 and n-6 PUFAs, particularly ALA and LA [82] exhibited increased levels of PUFAs after canning and storage in several studies [15,26,30,79]. Conversely, canning in OO, which is high in MUFAs, mainly OA [82], led to an increase in MUFA values in the flesh of canned fish samples [25,26,28,33,75,76,78]. SFO is a rich source of LA [82], thus, in the included studies, fish canned in SFO showed increased levels of PUFAs, mainly n-6, compared to initial values [13,26,37,76,77].

Given the wide variety of canned fish products, five studies have focused on evaluating changes in FA composition in specific fish species [15,30,68,75,80]. A study investigating the lipid content of two sardine species canned in either OO or TS found that while lipid levels remained stable in sardines canned in TS, the EPA and DHA content decreased in both sardine samples [78]. Similarly, Tarley et al. compared different brands of whole sardines and measured the highest levels of EPA and DHA in canned samples in TS [23]. TS offers a comparable protective benefit, resulting in higher concentrations of total n-3 PUFAs and the highest ratio compared to SO in sardines [23]. The consistent lipid content observed in sardines canned in TS after sterilization underscores its superior preservative effect against lipid exchange and dilution [57]. In a study exploring the impact of filling materials on the FA composition of canned tuna, Dantas et al. reported significant differences in the compositions of PUFAs, MUFAs, and SFAs among the brands [31]. In this review we also corroborated that FA profile of canned fish is affected by numerous factors, including the fish species and filling medium.

### 3.2. Effects of Canning on Nutritional Implications

In 2008, a study highlighted significant findings regarding PUFA levels in canned fish available in the Polish market, concluding that canned fish can be considered a good source of EPA and DHA. Researchers also noted that as little as 30 g of sardine and 45 g of other canned fish products meet the recommended daily amount for n-3 FAs [57]. Another study by Tarley et al. reported similar findings, showing that sardines canned in TS had higher total n-3 FA content than those canned in SO. The same study indicated that sardines canned in TS are acceptable sources of n-3 and n-6 FAs [23]. Additionally, less than one serving of various canned fish contained the required daily amount of EPA and DHA [45]. Moreover, some researchers have emphasized that despite the canning process having a minor impact on the flesh, canned fish may still provide the recommended daily intake of EPA and DHA [25,78]. Our results also suggest that, although the EPA and DHA values decreased in canned samples compared to fresh ones, these fish can still be considered good sources of n-3 LCPUFAs. The main advantage of canning is that it extends the shelf life of the fish and provides a reliable alternative source of EPA and DHA, even in areas far from the sea where access to fresh seafood is significantly more limited.

### 3.3. Effects of Processing and Storage

An extensive range of industrial processes is applied to canned fish products, including storage pre-treatments such as chilling and freezing, cooking, sterilization, and post-storage [22]. In this review, we found that most studies reported significantly lower SFA, MUFA, EPA, and DHA values after pre-treatment and canning, especially when vegetable oil was used as the filling medium. Conversely, studies using no medium or brine as the filling medium generally reported no significant changes in the FA values of fish flesh. Consequently, it is inevitable that fish are exposed to various conditions that may adversely affect their nutritional and sensory quality [31]. Nevertheless, a strong relationship between the canning process and FA composition has been documented in the literature [25,28,30,78]. Numerous studies have shown a sharp decline in PUFA content following the canning process due to these treatments [20,21,81]. Additionally, a substantial body of research has observed that raw fish samples are richer in n-3 PUFAs compared to canned ones at the end of the process [13,15,24,26,75]. These findings further support the understanding of FA content differences between fresh and canned fish.

Undoubtedly, canning serves as a crucial method for preserving fish [26]. This technique involves two thermal steps, pre-cooking and sterilization, which together aim to permanently deactivate both enzymes and microorganisms, allowing the heat-processed fish to be stored for an extended period [13,22]. In canned fish, a liquid medium, such as brine or vegetable oil, is typically used to facilitate heat transfer to the flesh of the fish [26]. Some studies have suggested that the filling medium may cause varying degrees of dilution, partial extraction of certain components, and different heat transfer dynamics in fish muscle [15,75]. In 2007, research indicated that thermal treatment during the sterilization process led to an increase in free fatty acid (FFAs) values due to oxidation [15]. Similarly, various sterilization methods had comparable effects, resulting in a slight rise in FFA levels [62]. Concerning the alteration of the FA profile, studies revealed that the pre-cooking process caused a significant loss of fat [25]. Similarly, another study showed that the cooking process primarily affected MUFA and n-6 PUFA values, significantly reducing their composition [81]. Additionally, several studies concluded that sterilization led to a notable decrease in EPA and DHA content [30,33,37]. Conversely, a study by Selmi and Sadok indicated that the total fat content of fresh sardines increased after the addition of OO and sterilization, due to the integration of OO into the muscle. However, the lipid content of sardines in TS remained unchanged [78]. These findings may account for the relatively strong correlation between the filling medium and the fish at any stage of the canning process. Consequently, more comprehensive studies have been recommended to assess the relative effects of different filling media (such as brine, OO, SFO, SO, and other vegetable oils) on the lipid quality of canned fish [21].

The existing literature on PUFAs is extensive, with a particular focus on the n-3 to n-6 ratio [81]. It is well established that this ratio is crucial in dietetics, as it plays a vital role in the balanced biosynthesis of eicosanoids that regulate inflammation in organisms [83,84]. Recent studies have clearly demonstrated a significant decline in the n-3 to n-6 PUFA ratio during the canning process and storage [30,80]. The nutritional ratio of n-3/n-6 PUFAs is highly sensitive to the filling medium [77]. The use of high-n-6 PUFA oils, particularly SFO, leads to a dramatic decrease in this ratio (e.g., in silver carp from 1.42 to 0.24 in SFO-canned samples), thereby undermining the fish’s inherent anti-inflammatory benefits [26]. In contrast, a study led by Ruiz-Roso et al. highlighted that over a 5-year storage period, the n-3/n-6 ratio increased in coating oil samples [75]. A recent study by Naseri et al. argued that after long-term storage, the highest n-3/n-6 ratio was found in canned fish samples in brine [26]. Similarly, Tarley et al. noted that canned fish samples in TS had a higher n-3/n-6 ratio than other samples [23]. The most effective strategy for maintaining the native n-3/n-6 ratio is using aqueous filling media. Brine-canned silver carp consistently retained the highest ratio compared to oil-packed counterparts [26]. Overall, these findings indicate that both the canning medium and storage conditions significantly influence the preservation of the nutritional quality of fish, particularly concerning the n-3/n-6 PUFA balance. Using aqueous media such as brine or TS appears to be the most effective approach for minimizing the degradation of beneficial n-3 PUFAs and maintaining the optimal ratio associated with health benefits.

Data from several studies indicate that variations in FA composition occur during different stages of storage in canned fish [25,81]. The findings by García-Arias et al. revealed a significant increase in the fat content of canned samples stored for one year compared to sterilized samples, attributed to the effects of the filling medium [30]. Similar results have been reported by other researchers [80]. However, some studies have highlighted a significant loss in n-3 PUFA levels in canned fish during storage [15,75]. Additionally, when comparing the filling oil used for canning with the initial oil, n-6 PUFA content was found to decrease while n-3 PUFA values increased [75]. Interestingly, there were no major fluctuations in the total fractions of SFAs, MUFAs, PUFAs, or n-3 PUFAs in canned fish after canning and short-term storage [24]. Moreover, while it has been recommended that fish canned in aqueous media like brine be consumed within six months due to the instability of n-3 PUFAs, fish canned in oil exhibit different lipid profiles over time. These results further support the understanding of the complex lipid interactions and bidirectional exchanges between fish muscle and the filling medium during the canning and storage period.

### 3.4. Nutritional Value of Canned Fish

In the studies examining the EPA and DHA content in tuna or sardines, we observed a decline in these n-3 LCPUFA values in fish. This decrease was most pronounced in fish stored in oil for extended periods. Traditional methods of reporting FA content, such as percentage composition or concentration (g/100 g wet weight), often indicate a significant drop in EPA and DHA content in the solid fish muscle, frequently exceeding 20% in oil-canned products. However, this apparent loss is misleading, as it primarily results from dilution and displacement due to oil absorption into the muscle, rather than thermal destruction [30]. Some researchers contend that expressing EPA and DHA content as a percentage (%) or concentration (g/100 g) does not accurately reflect the actual changes in FAs post-sterilization [13,37]. For instance, the percentage of EPA and DHA in the solid parts of oil-canned products was observed to decrease by more than 20%, mainly due to oil migration into the fish meat. However, True Retention Rate (TRR) calculations, which consider the mass balance of the entire can contents (fish plus liquid), reveal that the actual EPA and DHA losses in the whole canned food were relatively minor, not exceeding 7.5%. Despite minimal whole-product loss, the fish solids alone retained only (64.8–71.1%) of the original EPA and DHA content found in the steamed raw material [13]. Thus, a comprehensive interpretation requires examining the mass balance using TRR calculations. This quantitative evidence strongly suggests that the stability of n-3 PUFAs during thermal sterilization is high, and the primary factor driving changes in the fish muscle composition is the nutrient separation between phases. Furthermore, initial processing steps like steaming generally did not significantly affect the percentage composition of EPA and DHA within the fish lipid [13].

### 3.5. Strengths and Limitations

The primary strength of this review lies in its comprehensive literature search across five databases, evaluating articles based on predefined inclusion and exclusion criteria to ensure methodological rigor. To minimize bias, two authors independently reviewed the articles at all stages, with data extraction also verified by a second author. We concentrated on a relatively homogeneous topic, examining articles that focused on the FA profiles of both fish and the filling medium, which allowed us to gain detailed insights into the bidirectional FA exchange. The broad geographic and species coverage, encompassing varied fish types from nearly all over the world, enhances the broader applicability of our results. The qualitative synthesis of the data, organized by fish species, filling medium, and storage, provides a structured overview of the available data.

However, it is important to note that this study had several limitations. A key limitation is the substantial heterogeneity across the included studies in terms of fish species, sterilization protocols, storage duration, and filling medium, which prevented a quantitative synthesis of the data in the form of a meta-analysis. The restriction to English-language publications may have excluded potentially relevant non-English studies. Additionally, the variability in analytical methods and reporting across studies further limits comparability. This systematic review focused solely on the bidirectional interchange of FAs between fish and coating media, while other aspects such as lipid oxidation, sensory qualities or safety of canned fish products were not addressed.

## 4. Materials and Methods

### 4.1. Study Design and Protocol

This systematic review was conducted in accordance with the Preferred Reporting Items for Systematic Reviews and Meta-Analyses (PRISMA 2020) guidelines [85]. As the review focused on experimental and analytical investigations of canned fish rather than human participants or health-related outcomes, prospective registration was not necessary.

### 4.2. Search Strategy and Keywords

A systematic literature search was conducted to identify peer-reviewed studies investigating FA composition and the interaction between canned fish and their filling media. The databases PubMed (MEDLINE), Scopus, Web of Science, Wiley Online Library, and the Cochrane Library were searched from their inception until 31 July 2025. Additionally, a grey literature search was performed via Google Scholar. Both extensive electronic and manual search techniques were employed.

The search strategy combined MeSH terms and free-text keywords related to canned fish and fatty acids, including: (“canned fish” OR “canned seafood” OR “preserved fish” OR “fish canning”) AND (“fatty acids” OR “fatty acid composition” OR “polyunsaturated fatty acids” OR “PUFA” OR “EPA” OR “DHA” OR “essential fatty acids”) AND (“filling medium” OR “covering oil” OR “brine” OR “liquid medium” OR “coating media”). Boolean operators (AND, OR), truncation and wildcard symbols were applied to refine the search. To effectively combine search terms, the strategy was adapted for each database (Supplementary File S1). Reference lists of included studies and relevant reviews were manually screened to identify additional eligible articles. To maintain consistency, the search was limited to English-language publications for reasons of feasibility and interpretative accuracy.

### 4.3. Eligibility Criteria

Studies were included if they met the following criteria:

- Analyzed FA profiles in both fish tissue and filling media (e.g., oil, brine, TS) to highlight changes in the FA profile from raw/initial materials to the canned version.
- Investigated and reported changes in FA composition during any stage of the canning process (raw, pre-processed, or post-sterilization).
- Examined interactions or bidirectional exchanges of FAs between fish and coating media.
- Published in peer-reviewed journals in English at any time.

Exclusion criteria included:

- Conference abstracts, editorials, and non-peer-reviewed reports.
- Studies unrelated to canned fish or without FA outcome measures.
- Duplicate studies, inaccessible full texts, or non-English publications.

### 4.4. Study Selection

After duplicates were removed, two reviewers (ÖFK, HKK) independently screened the titles and abstracts of all retrieved records based on predefined inclusion criteria. This was followed by a full-text assessment of studies that were potentially eligible. Any discrepancies were resolved through discussion or by consulting a third reviewer (ÉS). A PRISMA flow diagram was used to summarize the selection process, detailing the number of records identified, screened, excluded, and included (Figure 1).

### 4.5. Data Extraction

Data were extracted by one reviewer (ÖFK) and subsequently verified for completeness, accuracy, and consistency by a second reviewer (HKK or OH) using a pre-piloted extraction form. The following variables were recorded:

- Study details (author, year, country)
- Fish species
- Type of filling or coating medium
- Processing and storage conditions or duration
- Study objectives
- Analytical methods for FA determination, such as gas chromatography (GC) and gas chromatography–mass spectrometry (GC–MS)
- Data on FA composition (total fat, SFA, MUFA, PUFA, EPA, DHA)
- Observed changes in FA profile
- Reported mechanisms of lipid exchange between fish and media

### 4.6. Quality Assessment

All included studies were critically evaluated for methodological transparency, including the description of sample origin, processing conditions, analytical method validity, and outcome completeness.

### 4.7. Data Synthesis

A qualitative synthesis of the included studies was performed because of significant heterogeneity among them regarding fish and coating materials, outcome measures, and storage durations. Consequently, pooling the data for meta-analysis was deemed inappropriate. The findings were organized based on the following criteria:

- Type of fish species and initial FA profile.
- Influence of filling/coating media on FA composition.
- Effect of thermal processing and storage on FA stability.
- Bidirectional lipid migration between fish and the media.

Additionally, we narratively synthesized the findings of the included studies to explore the nutritional implications associated with these compositional changes.

## 5. Conclusions

This systematic review revealed that the FA composition of canned fish is significantly affected by various factors, including fish species, filling medium, sterilization method, and storage duration. The canning process causes bidirectional migration of lipids between the fish muscle and filling medium: marine FAs, such as EPA and DHA, migrate into the coating oil, enhancing its nutritional value, whereas the fish absorb exogenous FAs from the filling medium, altering their FA composition to resemble that of the coating. Notably, n-3 LCPUFAs, such as EPA and DHA, are sensitive to canning and generally decrease in fish canned in vegetable oils, whereas aqueous media, such as brine and TS, better preserve these EFA and maintain a more favorable n-3/n-6 PUFA ratio. This review highlights the complexity of the factors influencing FA profiles in canned fish and recommends the use of aqueous packing media or consumption of covering oils to optimize nutritional benefits. Further standardized research is necessary to clarify the relative impact of these factors on FA alterations and enhance the nutritional evaluation of canned fish products. As the majority of fish species intended for canning are stored in frozen or refrigerated conditions in factories prior to processing, the impact of this pre-canning storage on the FA composition of the final product is a significant and largely unexplored topic that requires further research. Furthermore, because standard canning operations require adherence to strict thermal protocols, future research should systematically investigate the precise effects of varying F_0_ values, maximum temperatures, and processing times on the bidirectional lipid interchange. Evaluating emerging thermal technologies alongside standard retort processing will be essential to establish optimal sterilization conditions that guarantee microbiological safety without compromising EFA retention.

## Figures and Tables

**Table 1:** Characteristics of the included studies.

First Author, Publication Year	Country	Catch of the Fish	Subject (Type of Sample)		Aim of the Study
			Fish	Filling Medium	
Hale MB, 1983 [21]	USA	Gulf of Mexico (vicinity of Panama City, Florida)	Spanish Sardine (*Sardinella aurita*) Chub mackerel (*Scomber japonicus*) Thread herring (*Opisthonema oglinum*)	Brine (15.8% NaCl); SO	To assess the effects of canning and packing media on fatty acid and lipid class compositions.
Aubourg SP, 1990 [15]	Spain	Atlantic Ocean (43° N and 27° W)	Albacore tuna (*Thunnus alalunga*)	SO	To investigate the changes and interactions in the lipid content and FA composition of fish and filling oils during canning and storage.
Garcia-Arias MT, 1994 [30]	Spain	Atlantic Ocean (43° N and 27° W)	Albacore tuna (*Thunnus alalunga*)	SO	To evaluate how canning process and storage period affect the total fat content and FA composition of white tuna.
Medina I, 1995 [18]	Spain	Atlantic Ocean (Atlantic Albacore)	Albacore tuna (*Thunnus alalunga*)	Brine (2%); SO	To assess the influence of filling media on lipid modifications during the industrial canning processes.
Ruiz-Roso B, 1998 [75]	Spain	Sada (Spain)	Sardine (*Sardina pilchardus*)	OO	To investigate the impact of the maturation and canning processes on the FA composition and organoleptic qualities.
Rossi M, 2001 [34]	Italy	Adriatic Sea	Sardine (*Sardina pilchardus*)	OO	To evaluate the interchange between fish and the filling oil.
Selmi S, 2007 [78]	Tunisia	Sidi Daoud, Tunisia	Spanish sardine (*Sardinella aurita*) Sardine (*Sardina pilchardus*)	OO; TS	To identify the effects of the canning process on the lipid content and FA profile of sardine canned in different filling medias.
Rasmussen RS, 2008 [24]	USA	U.S. West Coast	Albacore tuna (*Thunnus alalunga*)	No filling medium	To assess the effects of canning and short-term storage on the FA profile of once-cooked, raw-packed tuna.
Selmi S, 2008 [25]	Tunisia	Sidi Daoud, Tunisia	Tuna (*Thunnus thynnus*) Sardine (*Sardina pilchardus*)	OO; TS	To investigate the effect of canning and storage on FA profiles and quality indicators of tuna and sardine canned in different filling media.
Siriamornpun S, 2008 [80]	Thailand	n.d. (supplied by Thai-Ruamsin Co. Ltd., Bangkok, Thailand)	Tuna	Brine (1%)	To evaluate the effect of storage time on the composition and concentration of lipids and FAs in canned tuna over a 12-month period.
Naseri M, 2011 [26]	Iran	Khuzestan	Silver carp (*Hypophthalmichthys molitrix*)	SFO; OO; SO; Brine (2%)	To investigate the influence of different filling media on the oxidation and lipid quality of canned silver carp.
Naseri M, 2012 [79]	Iran	Caspian Sea	Sprat (*Clupeonella cultriventris*)	Brine (2%); SO	To determine the effects of canning storage of 3 years period on FA composition.
Czerner M, 2015 [81]	Argentina	Mar del Plata Port (38° S, 57°33′ W)	Anchovy (*Engraulis anchoita*)	SO; SFO	To assess the effects of salting-ripening, canning and marinating processes on chemical composition and FA profile with emphasis on LCPUFAs
Mesias M, 2015 [33]	Spain	Pacific Ocean (Tuna) Mediterranean Sea (Sardine)	Yellowfin tuna (*Thunnus albacares*) Sardine (*Sardina pilchardus*)	Brine (3%); SFO; OO	To examine the effects of two different sterilization treatment named conventional and alternative on the FA composition of tuna and sardine.
Herawati ERN, 2016 [20]	Indonesia	Gunungkidul, Yogyakarta	Catfish (*Pangasius sutchi*)	Brine (1%, 2% and 3%); Water	To investigate the effect of different concentrations of brine on the nutrient content and FA profile of canned catfish.
Gómez-Limia L, 2020 [76]	Spain	River Ulla, Galicia, NW Spain	European eels (*Anguilla anguilla*)	OO; SFO	To investigate FA profile changes for each canning process steps and during storage.
Domiszewski Z, 2021a [13]	Poland	FAO 27 (The NE Atlantic): herring, mackerel FAO 27 IIId (Baltic Sea): sprat	Atlantic mackerel (*Scomber scombrus*) Atlantic herring (*Clupea harengus*) Baltic sprat (*Sprattus sprattus*)	SFO; TS	To evaluate the influence of the industrial sterilization process on the true retention rate of EPA and DHA content of canned oily fish species.
Domiszewski Z, 2021b [37]	Poland	Baltic Sea	Sprat (*Sprattus sprattus*)	SFO	To assess the impact of technological processes on the true retention of EPA and DHA as well as the physical characteristics
Bouriga N, 2022 [28]	Tunisia	Fresh sardines purchased from a local fish landing centre (Bizerte, NE Tunisia)	Sardine (*Sardina pilchardus*)	OO; GSO	To determine whether grapeseed oil, rich in polyphenols and PUFAs, could improve the nutritional quality and oxidative stability compared to olive oil.
Prego R, 2022 [77]	Spain	Vigo, NW Spain	Atlantic mackerel (*Scomber scombrus*)	Water; Brine (2%); SFO; Refined OO; EVOO	To evaluate the influence of prior frozen storage on canned fish FA composition in a wide range of filling media

Abbreviations: DHA: docosahexaenoic acid; EPA: eicosapentaenoic acid, EVOO: extra virgin olive oil; FA: fatty acid; GSO: grapeseed oil; LCPUFA: long-chain polyunsaturated fatty acid; n.d.: no data; N: north; NE: northeast; NW: northwest; OO: olive oil; PUFA: polyunsaturated fatty acid; S: south; SFO: sunflower oil; SO: soybean oil; TS: tomato sauce; W: west.

**Table 2:** Changes in the fatty acid composition of canned fish in different species compared to the initial (raw) samples and role of filling media, type of sterilization and/or storage time.

Type of Fish		Filling Medium	Outcomes	Ref.
Tuna species	Albacore tuna (*Thunnus alalunga*)	SO	Compared to initial (cooked only) samples: SFA ↓#, MUFA ↓#, PUFA ↑#, EPA ↓#, DHA ↓#	[15]
		SO	Compared to raw samples (stored for 3 years): Sterilized 55 min: SFA ↓#, MUFA ↑#, PUFA ↑#, EPA ↓*, DHA ↓* Sterilized 90 min: SFA ↓#, MUFA ↑#, PUFA ↑#, EPA ↓*, DHA ↓*	[30]
		SO	Compared to raw samples: total n-3 PUFA ↓*	[18]
		No filling medium	Compared to raw samples (after 5 weeks storage): SFA → &, MUFA → &, PUFA → &, EPA → &, DHA → &	[24]
		Brine (2%)	Compared to raw samples: total n-3 PUFA ↓*	[18]
	Yellowfin tuna (*Thunnus albacares*)	Brine (3%)	Compared to retort sterilization, high pressure thermal sterilization: SFA → &, MUFA → &, PUFA → &, EPA → &, DHA → &	[33]
		SFO	Compared to retort sterilization, high pressure thermal sterilization: SFA ↓*, MUFA → &, PUFA → &, EPA → &, DHA → &	[33]
	Bluefin tuna (*Thunnus thynnus*)	OO	Compared to raw samples (after 6 months storage): SFA ↓*, MUFA ↑*, PUFA ↓*, EPA ↓*, DHA ↓*	[25]
	Tuna (Unspecified)	Brine (1%)	Compared to the initial time point (after 12 months storage): SFA ↑*, MUFA ↑*, PUFA ↓*, EPA ↓*, DHA ↓*	[80]
Sardines and Sardinellas	Sardine (*Sardina pilchardus*)	OO	Compared to raw samples: After 6 months storage: SFA ↓, MUFA ↑, n-3 PUFA ↓, n-6 PUFA ↑, EPA ↓, DHA ↑ After 5 years storage: SFA ↓, MUFA ↑, n-3 PUFA ↓, n-6 PUFA ↑, EPA ↓, DHA ↑	[75]
		OO	Compared to retort sterilization, high pressure thermal sterilization: SFA → &, MUFA ↑*, PUFA ↓*, EPA → &, DHA ↓*	[33]
		OO	Compared to raw samples (after 3 months storage): SFA ↓*, MUFA ↑*, PUFA ↑*, EPA ↓*, DHA → &	[28]
		TS	Compared to raw samples: SFA ↓*, MUFA ↑*, PUFA → &, EPA ↓*, DHA ↓*	[78]
		TS	Compared to raw samples (after 6 months storage): SFA ↓*, MUFA ↑*, PUFA → &, EPA ↓*, DHA ↓*	[25]
		GSO	Compared to raw samples (after 3 months storage): SFA ↓*, MUFA ↑*, PUFA ↑*, EPA ↓*, DHA ↓*	[28]
	Spanish sardine (*Sardinella aurita*)	Brine (2%)	Compared to raw samples: SFA → #, MUFA → #, PUFA → #, EPA → #, DHA → #	[21]
		SO	Compared to raw samples: SFA ↓#, MUFA ↓#, EPA ↓#, DHA ↓#	[21]
		OO	Compared to raw samples: SFA ↓*, MUFA ↑*, PUFA → &, EPA ↓*, DHA ↓*	[78]
Mackerels	Atlantic mackerel (*Scomber scombrus*)	TS	Compared to raw samples: SFA → &, MUFA → &, PUFA ↑*, EPA → &, DHA → &	[13]
		SFO	Compared to raw samples: SFA ↓*, MUFA → &, PUFA ↑*, EPA ↓*, DHA ↓*	[13]
		SFO	Compared to raw samples: SFA ↓*, MUFA ↓*, PUFA ↑*, EPA ↑*, DHA ↑*	[77]
		Water	Compared to raw samples: SFA ↓*, MUFA → &, PUFA ↑*, EPA ↑*, DHA → &	[77]
		Brine (2%)	Compared to raw samples: SFA ↓*, MUFA → &, PUFA ↑*, EPA ↑*, DHA → &	[77]
		Refined OO	Compared to raw samples: SFA ↓*, MUFA → &, PUFA ↑*, EPA → &, DHA ↑*	[77]
		EVOO	Compared to raw samples: SFA ↓*, MUFA → &, PUFA ↑*, EPA → &, DHA ↑*	[77]
	Chub mackerel (*Scomber japonicus*)	Brine (2%)	Compared to raw samples: SFA → #, MUFA → #, PUFA → #, EPA → #, DHA → #	[21]
Sprat	Baltic sprat (*Sprattus sprattus*)	SFO	Compared to raw samples: SFA ↓*, MUFA ↓*, PUFA ↑*, EPA ↓*, DHA ↓*	[13]
		SFO	Compared to raw samples: SFA ↓*, MUFA ↓*, PUFA ↑*, EPA ↓*, DHA ↓*	[37]
		TS	Compared to raw samples: SFA ↓*, MUFA → &, PUFA → &, EPA → &, DHA → &	[13]
	Sprat (*Clupeonella cultriventris*)	Brine (2%)	Compared to raw samples: SFA ↑*, MUFA ↓*, PUFA → &, EPA ↓*, DHA → &	[79]
		SO	Compared to raw samples: SFA ↓*, MUFA ↓*, PUFA ↑*, EPA ↓*, DHA ↓*	[79]
Herring	Atlantic herring (*Clupea harengu*)	SFO	Compared to raw samples: SFA ↓*, MUFA → &, PUFA ↑*, EPA ↓*, DHA ↓*	[13]
		TS	Compared to raw samples: SFA → &, MUFA → &, PUFA → &, EPA → &, DHA → &	[13]
	Thread herring (*Opisthonema oglinum*)	Brine (2%)	Compared to raw samples: SFA → #, MUFA → #, PUFA ↑#, EPA → #, DHA → #	[21]
Anchovy	Anchovy (*Engraulis anchoita*)	SO	Compared to raw samples: SFA → &, MUFA ↓*, PUFA → &, EPA → &, DHA → &	[81]
		SFO	Compared to raw samples (9 days marinated in SFO): SFA → &, MUFA → &, PUFA → &, EPA → &, DHA ↑*	[81]
Freshwater and Diadromous Species	European eel (*Anguilla anguilla*)	OO	Compared to raw samples (after 12 months storage): SFA ↓*, MUFA ↑*, PUFA ↓*, EPA ↓*, DHA ↓* Compared to raw samples (after 12 months storage in SOO): SFA ↓*, MUFA ↑*, PUFA ↓*, EPA ↓*, DHA ↓*	[76]
		SFO	Compared to raw samples (after 12 months storage): SFA ↓*, MUFA ↓*, PUFA ↑*, EPA ↓*, DHA ↓*	[76]
	Silver carp (*Hypophthalmichthys molitrix*)	SFO	Compared to raw samples (after 4 months storage): SFA ↓*, MUFA ↓*, PUFA ↑*, EPA → &, DHA ↓*	[26]
		OO	Compared to raw samples (after 4 months storage): SFA ↓*, MUFA ↑*, PUFA → &, EPA → &, DHA → &	[26]
		SO	Compared to raw samples (after 4 months storage): SFA ↓*, MUFA ↓*, PUFA ↑*, EPA → &, DHA → &	[26]
		Brine (2%)	Compared to raw samples (after 4 months storage): SFA ↓*, MUFA ↓*, PUFA → &, EPA ↑*, DHA → &	[26]
	Catfish (*Pangasius sutchi*)	Brine (3%)	Compared to raw samples: SFA ↑#, MUFA ↑#, PUFA ↑#, EPA ↑#, DHA ↑# Compared to catfish canned in water, canned catfish in brine (3%): SFA ↓#, MUFA ↓#, PUFA ↓#, EPA ↓#, DHA ↓#”	[20]
		Water	Compared to raw samples: SFA ↑#, MUFA ↑#, PUFA ↑#, EPA ↑#, DHA ↑#	[20]

Abbreviations: DHA: docosahexaenoic acid; EPA: eicosapentaenoic acid, EVOO: extra virgin olive oil; GSO: grapeseed oil; m: month; MUFA: monounsaturated fatty acid, OO: olive oil; PUFA: polyunsaturated fatty acid; SFA: saturated fatty acid; SFO: sunflower oil; SO: soybean oil; TS: tomato sauce; #: statistical significance was not reported, &: no statistical significance, *: statistical significance (*p* < 0.05), ↓: increased; ↑: decreased; →: no change.

**Table 3:** Changes in the fatty acid composition of filling medium in different fish species compared to the initial medium samples and role of type of sterilization and/or storage time.

Filling Medium	Type of Fish	Change in Filling Medium FA Composition	Ref.
OO	European eel (*Anguilla anguilla*)	compared to raw OO (after 12 months storage): SFA ↓*, MUFA ↑*, PUFA ↓*, EPA → &, DHA ↑* compared to raw SOO (after 12 months storage): SFA ↓*, MUFA ↑*, PUFA ↓*, EPA ↓*, DHA ↑*	[76]
	Sardine (*Sardina pilchardus*)	Compared to initial OO: After 6 months storage: SFA ↑, MUFA ↓, n-3 PUFA ↑, n-6 PUFA ↓, EPA ↑, DHA ↑ After 5 years storage: SFA ↑, MUFA ↓, n-3 PUFA ↑, n-6 PUFA ↑, EPA ↑, DHA ↑	[75]
		Compared to native OO (after 120 days storage): SFA ↑#, MUFA ↓#, PUFA ↑#, EPA ↑#, DHA ↑#	[34]
	Silver carp (*Hypophthalmichthys molitrix*)	SFA → &, MUFA ↓*, PUFA → &, EPA ↑+, DHA ↑+	[26]
SFO	Atlantic herring (*Clupea harengu*)	SFA ↑, MUFA ↑, PUFA ↓, EPA ↑, DHA ↑ (reported as O or TS) ş	[13]
	Atlantic mackerel (*Scomber scombrus*)	SFA ↑, MUFA ↑, PUFA ↓, EPA ↑, DHA ↑ (reported as O or TS) ş	[13]
	Baltic sprat (*Sprattus sprattus*)	SFA ↑, MUFA ↑, PUFA ↓, EPA ↑, DHA ↑ (reported as O or TS) ş	[13]
		fresh/smoked liquid parts (oil + water after sterilization) compared to initial oil: SFA → &, MUFA → &, PUFA → &, EPA ↑+, DHA ↑+ frozen/smoked liquid parts (oil + water after sterilization) compared to initial oil: SFA ↑*, MUFA → &, PUFA → &, EPA ↑+, DHA ↑+”	[37]
	European eel (*Anguilla anguilla*)	compared to raw SFO (after 12 months storage): SFA ↑*, MUFA → &, PUFA ↓*, EPA ↓*, DHA ↑+	[76]
	Silver carp (*Hypophthalmichthys molitrix*)	In SFO: SFA ↑*, MUFA ↑*, PUFA ↓*, EPA ↑+, DHA ↑+	[26]
SO	Albacore tuna (*Thunnus alalunga*)	In SO: EPA ↑#, DHA ↑#	[15]
		SO (after 3 years storage) compared to sterilization at 55 min, 90 min, respectively: Sterilized 55 min: EPA →, DHA → Sterilized 90 min: EPA →, DHA ↓*	[30]
	Sprat (*Clupeonella cultriventris*)	In SO: SFA ↑*, MUFA → &, PUFA ↓*, EPA ↑+, DHA ↑+	[79]
	Silver carp (*Hypophthalmichthys molitrix*)	In SO: SFA ↑*, MUFA → &, PUFA ↓*, EPA ↑+, DHA ↑+	[26]
TS	Atlantic herring (*Clupea harengu*)	In TS: SFA ↑, MUFA ↑, PUFA ↓, EPA ↑, DHA ↑ (reported as O/TS) ş	[13]
	Atlantic mackerel (*Scomber scombrus*)	In TS: SFA ↑, MUFA ↑, PUFA ↓, EPA ↑, DHA ↑ (reported as O/TS) ş	[13]
	Baltic sprat (*Sprattus sprattus*)	In TS: SFA ↑, MUFA ↑, PUFA ↓, EPA ↑, DHA ↑ (reported as O/TS) ş	[13]

Abbreviations: DHA: docosahexaenoic acid; EPA: eicosapentaenoic acid, EVOO: extra virgin olive oil; FA: fatty acid; GSO: grapeseed oil; m: month; MUFA: monounsaturated fatty acid, OO: olive oil; PUFA: polyunsaturated fatty acid; SFA: saturated fatty acid; SFO: sunflower oil; SO: soybean oil; TS: tomato sauce; #: statistical significance was not reported, &: no statistical significance, *: statical significance (*p* < 0.05), ş: it was noted that pre-sterilization fatty acid profile of the tomato sauce was similar to sunflower oil (due to 2–2.5% sunflower oil content); +: not detected in the initial or raw sample ↓: increased; ↑: decreased; →: no change.

**Table 4:** EPA and DHA levels (in *w*/*w*%) and their percentage change in sardine and tuna samples as a result of canning and storage.

Type of Fish	Filling Medium	Storage Time	EPA, Raw	EPA, Canned	EPA Change	DHA, Raw	DHA, Canned	DHA Change	Ref.
*Sardina pilchardus*	OO	n/a (retort sterilization)	2.60	2.60	0.00%	3.80	3.80	0.00%	[33]
		n/a (HP thermal sterilization)	2.60	1.70	−34.6%	3.80	1.60	−57.9%	
		1 month	6.13	3.78	−38.3%	27.41	31.59	+15.2%	[28]
		3 months	6.13	3.22	−47.5%	27.41	28.37	+3.5%	
		12 months	11.48	6.60	−42.5%	5.53	6.29	+13.7%	[75]
		5 years	11.48	7.83	−31.8%	5.53	6.68	+20.8%	
	GSO	1 month	6.13	3.18	−48.1%	27.41	25.52	−6.9%	[28]
		3 months	6.13	1.69	−72.4%	27.41	20.95	−23.6%	
	TS	n/a	6.24	4.15	−33.5%	33.61	24.79	−26.2%	[78]
		6 months	6.24	3.92	−37.2%	33.61	26.31	−21.7%	[25]
Albacore tuna (*Thunnus alalunga*)	no medium	0 day	9.10	9.10	0.0%	33.80	33.70	−0.3%	[24]
		5 weeks	9.10	9.10	0.0%	33.80	33.20	−1.8%	
	no medium (belly flap)	7 months	5.10	3.20	−37.3%	15.40	13.10	−14.9%	[15]
	no medium (back muscle)	11 months	5.40	3.70	−31.5%	20.20	15.30	−23.5%	
	no medium (ventral muscle	11 months	5.20	2.70	−48.1%	20.20	12.20	−39.6%	
	SO	0 day (55 min sterilization)	8.30	4.70	−43.4%	28.70	15.50	−46.0%	[30]
		0 day (90 min sterilization)	8.30	3.90	−53.0%	28.70	11.30	−60.6%	
		3 years (55 min sterilization)	8.30	3.00	−63.9%	28.70	9.30	−67.6%	
		3 years (90 min sterilization)	8.30	3.70	−55.4%	28.70	11.50	−59.9%	

Abbreviations: DHA: docosahexaenoic acid; EPA: eicosapentaenoic acid, GSO: grapeseed oil; n/a: not applicable; OO: olive oil; SO: soybean oil; TS: tomato sauce.

## Data Availability

No new data were created or analyzed in this study. Data sharing is not applicable to this article.

## Supplementary Materials

The following supporting information can be downloaded at: https://www.mdpi.com/article/10.3390/md24060204/s1, Supplementary File S1: Search strategies; Supplementary File S2: Characteristics of the excluded studies [11,12,21,22,23,27,29,31,39,40,41,42,43,44,45,46,47,48,49,50,51,52,53,54,55,56,57,58,59,60,61,62,63,64,65,66,67,68,69,70,71,72,73,86].; Supplementary File S3: Characteristics of included studies; Supplementary File S4: PRISMA Checklist [13,15,18,20,21,24,25,26,28,30,33,34,37,75,76,77,78,79,80,81,87].

## Abbreviations

The following abbreviations are used in this manuscript:

AHA	American Heart Association
ALA	Alpha-linolenic acid
DHA	Docosahexaenoic acid
EFA	Essential fatty acids
EPA	Eicosapentaenoic acid
EVOO	Extra virgin olive oil
FAs	Fatty acids
FFA	Free fatty acids
GC	Gas Chromatography
GC–MS	Gas Chromatography–Mass Spectrometry
GSO	Grapeseed oil
LA	Linoleic acid
LCPUFA	Long-chain polyunsaturated fatty acids
MUFA	Monounsaturated fatty acids
n-3	Omega-3
n-6	Omega-6
OA	Oleic acid
OO	Olive oil
PRISMA	Preferred Reporting Items for Systematic Reviews and Meta-Analyses
PUFAs	Polyunsaturated fatty acids
SFA	Saturated fatty acids
SFO	Sunflower oil
SO	Soybean oil
TRR	True retention rate
TS	Tomato sauce
WHO	World Health Organization
