# Supplementary material for: Systematic Review of Fatty Acid Composition and the Influence of Coating Media on Fatty Acid Profiles in Canned Fish
Source: Mar Drugs. 2026 Jun 10;24(6):204. doi: 10.3390/md24060204 (PMC13302670; doi:10.3390/md24060204)
Supplement: Supplementary file 1 [file marinedrugs-24-00204-s001.zip › SupplFiles/Supplementary file S2_ excluded studies.pdf]

Supplementary file S2: Characteristics of excluded studies

1	Fuadi et al. (2014)	Wrong outcome
2	Carvalho et al. (2018)	Wrong outcome
3	Bogdanović, Šimat, et al. (2014)	Wrong publication type
4	Lapis et al. (2013)	Wrong study design
5	Kwang-Soo Oh et al. (1989)	Wrong language
6	El-Lahamy AA, Mohamed HR. (2020)	Wrong publication type
7	Dantas et al. (2021)	Wrong study design
8	Caponio et al. (2011)	Wrong study design
9	Tarley CR et al. (2004)	Wrong study design
10	Caponio F et al. (2003)	Wrong outcome
11	Gladyshev MI et al. (2009)	Wrong study design
12	Miklavčič A et al. (2011)	Wrong outcome
13	Aubourg S et al (1997)	Wrong outcome
14	Usydus Z et al. (2008)	Wrong study design
15	Aubourg SP et al (1995)	Wrong language
16	Malcolm B. Hale, Thomas Brown. (1983)	Wrong outcome
17	Aubourg SP. (2001)	Wrong publication type
18	Singer P, et al. (2021)	Wrong publication type
19	Budiadnyani IG et al. (2024)	Wrong outcome
20	Song G et al. (2018)	Wrong study design
21	Mahmoodi V et al. 2017	Wrong outcome
22	Nazari B et al. (2008)	Wrong study design
23	Nava V et al. (2023)	Wrong study design
24	Shim SM et al. (2004)	Wrong outcome
25	Kołakowska A, et al. (2002)	Wrong outcome
26	Medina I et al. (2000)	Wrong study design
27	Villamarín E et al. (2023)	Wrong outcome
28	Mkadem H et al. (2019)	Wrong study design
29	Sirot V et al. (2008)	Wrong study design
30	Peycheva K et al. (2024)	Wrong study design
31	Alloyarova Y et al. (2019)	Wrong publication type
32	Aubourg SP. (2023)	Wrong publication type
33	Raghavan S, Kristinsson HG. (2014)	Wrong publication type
34	Ortiz J et al. (2014)	Wrong study design
35	Medina I et al. (1998)	Wrong outcome
36	Barbosa RG et al (2018)	Wrong outcome
37	Malga JM et al. (2022)	Wrong study design
38	Suárez-Medina MD et al. (2024)	Wrong publication type
39	Barbosa RG et al (2019)	Wrong study design
40	Esfahani ST et al. (2024)	Wrong outcome
41	Medina I et al. (1999)	Wrong study design
42	Méndez L et al. (2022)	Wrong outcome
43	Aubourg SP et al (2020)	Wrong study design
44	Proctor BE et al. (1961)	Wrong outcome

References

1. Fuadi I, Suseno SH, Ibrahim B. Characterization of fish oil from mackerel (Scomber japonicus) canning by product. Asian Journal of Agriculture and Food Sciences. 2014 Jun 15;2(3).
2. Carvalho AP, Amorim M, Rodriguez-Alcala L, Fontecha J, Castro PM, Pintado ME. Sardine canning byproducts as sources of functional ingredients. ACS Sustainable Chemistry & Engineering. 2018 Sep 12;6(11):15447-54.
3. Bogdanović T, Šimat V, Pleadin J, Vulić A, Petričević S, Blažić M. Proximate composition, fatty acids profile and nutriotional value of different canned sardines produced in Croatia. InBook of abstracts: 8th International Congress of Food Technologists, Biotechnologists and Nutritionists 2014 (p. 146).
4. Lapis TJ, Oliveira AC, Crapo CA, Himelbloom B, Bechtel PJ, Long KA. Supplementing long-chain n-3 polyunsaturated fatty acids in canned wild P acific pink salmon with A laska salmon oil. Food Science & Nutrition. 2013 Jan;1(1):15-26.
5. Oh KS, Kim JG, Sung DW, Lee HJ, Lee EH. Fatty acid composition of canned fish and shellfish products on Korean market. Journal of the Korean Society of Food Science and Nutrition. 1989;18(2):211-5.
6. El-Lahamy AA, Mohamed HR. Changes in fish quality during canning process and storage period of canned fish products. Journal of Nutritional Dietetics & Probiotics. 2020;3(1):180024.
7. Dantas NM, de Oliveira VS, Sampaio GR, Chrysostomo YS, Chávez DW, Gamallo OD, Sawaya AC, da Silva Torres EA, Saldanha T. Lipid profile and high contents of cholesterol oxidation products (COPs) in different commercial brands of canned tuna. Food Chemistry. 2021 Aug 1;352:129334.
8. Caponio F, Summo C, Pasqualone A, Gomes T. Fatty acid composition and degradation level of the oils used in canned fish as a function of the different types of fish. Journal of Food Composition and Analysis. 2011 Dec 1;24(8):1117-22.
9. Tarley CR, Visentainer JV, Matsushita M, de Souza NE. Proximate composition, cholesterol and fatty acids profile of canned sardines (Sardinella brasiliensis) in soybean oil and tomato sauce. Food chemistry. 2004 Nov 1;88(1):1-6.
10. Caponio F, Gomes T, Summo C. Quality assessment of edible vegetable oils used as liquid medium in canned tuna. European Food Research and Technology. 2003 Feb;216(2):104-8.
11. Gladyshev MI, Sushchik NN, Makhutova ON, Kalachova GS. Content of essential polyunsaturated fatty acids in three canned fish species. International journal of food Sciences and Nutrition. 2009 Jan 1;60(3):224-30.
12. Miklavčič A, Stibilj V, Heath E, Polak T, Tratnik JS, Klavž J, Mazej D, Horvat M. Mercury, selenium, PCBs and fatty acids in fresh and canned fish available on the Slovenian market. Food Chemistry. 2011 Feb 1;124(3):711-20.
13. Aubourg S, Gallardo JM, Medina I. Changes in lipids during different sterilizing conditions in canning albacore (Thunnus alalunga) in oil. International journal of food science & technology. 1997 Oct;32(5):427-31.
14. Usydus Z, Szlinder-Richert J, Polak-Juszczak L, Kanderska J, Adamczyk M, Malesa-Ciecwierz M, Ruczynska W. Food of marine origin: between benefits and potential risks. Part I. Canned fish on the Polish market. Food Chemistry. 2008 Dec 1;111(3):556-63.
15. Aubourg SP, Medina I, Gallardo JM, Pérez Martín RI. Efecto del enlatado en aceite y salmuera y su posterior almacenamiento sobre los lípidos de la bacoreta (Euthynnus alletteratus). 1995
16. Malcolm B. Hale, Thomas Brown. Fatty acids and lipid classes of three underutilized species and changes due to canning. Marine Fisheries Review. 1983;45:45.
17. Aubourg SP. Loss of quality during the manufacture of canned fish products. Food Science and Technology International. 2001 Jun;7(3):199-215.
18. Singer P, Richter V, Singer K, Löhlein I. Analyses and declarations of omega-3 fatty acids in canned seafood may help to quantify their dietary intake. Nutrients. 2021 Aug 26;13(9):2970.

19. Budiadnyani IG, Dewi RN, Panjaitan FC, Sayuti M. Physicochemical and Fatty Acid Profile of Refined Tuna Fish Oil By-Product from Canning and Meal Fish Industries. Trends in Sciences. 2024 Feb 25;21(5):7380-.
20. Song G, Dai Z, Shen Q, Peng X, Zhang M. Analysis of the changes in volatile compound and fatty acid profiles of fish oil in chemical refining process. European Journal of Lipid Science and Technology. 2018 Feb;120(2):1700219.
21. Mahmoodi, V., J. Keramat, M. Hojjatoleslamy, and H. Molavi. "Fatty acid profile and Quality of the oil extracted from Tuna Fish (Katsuwonus pelamis, Thunnus albacares) precooking liquid waste in canning factorys." (2017): 293-304.
22. Nazari B, Asgary S, Sarafzadegan N, Saberi S, Mohammadifard N. Content of long chain omega-3 fatty acid composition in some Iranian canned fish. ARYA Atherosclerosis Journal 2008, 4(3): 93-97
23. Nava V, Turco VL, Licata P, Panayotova V, Peycheva K, Fazio F, Rando R, Di Bella G, Potortì AG. Determination of fatty acid profile in processed fish and shellfish foods. Foods. 2023 Jul 7;12(13):2631.
24. Shim SM, Dorworth LE, Lasrado JA, Santerre CR. Mercury and fatty acids in canned tuna, salmon, and mackerel. Journal of Food Science. 2004 Dec;69(9):C681-4.
25. Kołakowska A, Stypko K, Domiszewski Z, Bienkiewicz G, Perkowska A, Witczak A. Canned cod liver as a source of n-3 polyunsaturated fatty acids, with a reference to contamination. Food/Nahrung. 2002 Jan 1;46(1):40-5.
26. Medina I, Sacchi R, Aubourg S. Application of 13C NMR to the selection of the thermal processing conditions of canned fatty fish. European Food Research and Technology. 2000 Jan;210(3):176-8.
27. Villamarín E, Martínez B, Trigo M, Aubourg SP. Influence of different previous frozen holding periods on the canned fish quality. Foods. 2023 Nov 13;12(22):4117.
28. Mkadem H, Kaanane A. Recovery and characterization of fish oil from by-products of sardine (Sardina pilchardus) in the canning process. Journal of Aquatic Food Product Technology. 2019 Nov 26;28(10):1037-50.
29. Sirot V, Oseredczuk M, Bemrah-Aouachria N, Volatier JL, Leblanc JC. Lipid and fatty acid composition of fish and seafood consumed in France: CALIPSO study. Journal of food composition and analysis. 2008 Feb 1;21(1):8-16.
30. Peycheva K, Panayotova V, Hristova T, Merdzhanova A, Dobreva D, Stoycheva T, Stancheva R, Licata P, Fazio F. Metal Content, Fatty Acid and Vitamins in Commercially Available Canned Fish on the Bulgarian Market: Benefit–Risk Ratio Intake. Foods. 2024 Mar 19;13(6):936.
31. Alloyarova Y, Alloyarov K, Kuranova L, Karpovich I. Quality assessment of the canned fish made of capelin of the prolonged storage period produced by the soft conditions of fish smoking. InIOP Conference Series: Earth and Environmental Science 2019 Jul 1 (Vol. 302, No. 1, p. 012014). IOP Publishing.
32. Aubourg SP. Enhancement of lipid stability and acceptability of canned seafood by addition of natural antioxidant compounds to the packing medium—A review. Antioxidants, 12 (2), 245 [Internet]. 2023
33. Raghavan S, Kristinsson HG. Influence of processing on lipids and lipid oxidation in aquatic foods. Antioxidants and functional components in aquatic foods. 2014 Jun 2:43-94.
34. Ortiz J, Vivanco JP, Aubourg SP. Lipid and sensory quality of canned Atlantic salmon (Salmo salar): Effect of the use of different seaweed extracts as covering liquids. European Journal of Lipid Science and Technology. 2014 May;116(5):596-605.
35. Medina I, Sacchi R, Biondi L, Aubourg SP, Paolillo L. Effect of packing media on the oxidation of canned tuna lipids. Antioxidant effectiveness of extra virgin olive oil. Journal of Agricultural and Food Chemistry. 1998 Mar 16;46(3):1150-7.

36. Barbosa RG, Trigo M, Fett R, Aubourg SP. Impact of a packing medium with alga Bifurcaria bifurcata extract on canned Atlantic mackerel (Scomber scombrus) quality. Journal of the Science of Food and Agriculture. 2018 Jul;98(9):3462-7.
37. Malga JM, Trigo M, Martínez B, Aubourg SP. Preservative effect on canned mackerel (Scomber colias) lipids by addition of octopus (Octopus vulgaris) cooking liquor in the packaging medium. Molecules. 2022 Jan 24;27(3):739.
38. Suárez-Medina MD, Sáez-Casado MI, Martínez-Moya T, Rincón-Cervera MÁ. The effect of low temperature storage on the lipid quality of fish, either alone or combined with alternative preservation technologies. Foods. 2024 Apr 3;13(7):1097.
39. Barbosa RG, Trigo M, Campos CA, Aubourg SP. Preservative Effect of Algae Extracts on Lipid Composition and Rancidity Development in Brine-Canned Atlantic Chub Mackerel (Scomber colias). European Journal of Lipid Science and Technology. 2019 Aug;121(8):1900129.
40. Esfahani ST, Zamindar N, Esmaeili Y, Sharifian S. Effect of initial quality of oil and thermal processing on oxidation indexes in canned tuna. Applied Food Research. 2024 Dec 1;4(2):100553.
41. Medina I, Satué-Gracia MT, German JB, Frankel EN. Comparison of natural polyphenol antioxidants from extra virgin olive oil with synthetic antioxidants in tuna lipids during thermal oxidation. Journal of agricultural and food chemistry. 1999 Dec 20;47(12):4873-9.
42. Méndez L, Trigo M, Zhang B, Aubourg SP. Antioxidant Effect of Octopus Byproducts in Canned Horse Mackerel (Trachurus trachurus) Previously Subjected to Different Frozen Storage Times. Antioxidants. 2022 Oct 23;11(11):2091.
43. Aubourg SP, Trigo M, Martínez B, Rodríguez A. Effect of prior chilling period and alga-extract packaging on the quality of a canned underutilised fish species. Foods. 2020 Sep 21;9(9):1333.
44. Proctor BE, Miller SA, Goldblith SA, Wick EL, PARTSER E, Sapers GM, Solberg M. The Nutritive Value of Maine Sardines. a: 1. Chemical Composition. Journal of Food Science. 1961 May;26(3):283-7.
