# Supplementary material for: Systematic Review of Fatty Acid Composition and the Influence of Coating Media on Fatty Acid Profiles in Canned Fish
Source: Mar Drugs. 2026 Jun 10;24(6):204. doi: 10.3390/md24060204 (PMC13302670; doi:10.3390/md24060204)
Supplement: Supplementary file 1 [file marinedrugs-24-00204-s001.zip › SupplFiles/Supplementary file S3_Characteristics of included studies.pdf]

	Description	
**Study Characteristics**	**Author, date**	Hale, M. B., & Brown, T. (1983)
	**Country**	USA
	**Publication type**	Full text - Journal Article (*Marine Fisheries Review*)
	**Funding**	National Marine Fisheries Service, NOAA
	**Conflict**	None explicitly declared
**Material & Methods**	**Aim of study**	To present fatty acid, lipid class, and proximate composition data on three underutilized species and indicate whether nutritionally desirable polyunsaturated fatty acids are affected by the canning process
	**Geographical area of catch**	Gulf of Mexico (vicinity of Panama City, Florida)
	**Fish type**	Spanish Sardine (*Sardinella aurita*), Chub mackerel (*Scomber japonicus*), Thread herring (*Opisthonema oglinum*)
	**Filling medium**	Brine (2%),<br>Soybean oil (SO) (tested on Spanish sardine only)
	**Study design**	Fish were dressed, brined (15.8% NaCl), packed, steam precooked (190°F), drained, and filled with hot brine (or soybean oil for specific sardine samples). Cans were sterilized (Fo = 12) and drained solids were analyzed
	**Duration of the study**	Analyzed after canning (specific storage duration not defined)
	**FA analysis**	Gas Chromatography (GC) using a 50 m flexible fused silica capillary column coated with Carbowax 20-M.
**Outcomes**	**Brine Packing:** Compared to raw samples, heat processing in sealed cans had no significant effect on the fatty acid profile or lipid class composition for any of the three species.<br>◦ **Spanish Sardine:** SFA (37.7% vs 37.3%), MUFA (19.4% vs 17.8%), PUFA (42.9% vs 44.9%).<br>◦ **Chub Mackerel:** SFA (34.8% vs 34.2%), MUFA (22.5% vs 19.0%), PUFA (42.7% vs 46.9%).<br>◦ **Thread Herring:** SFA (43.2% vs 42.0%), MUFA (19.2% vs 17.5%), PUFA (37.6% vs 40.5%).<br>• **Soybean Oil Packing (Spanish Sardine):** The use of vegetable oil had major effects on the product fatty acid profile.<br>◦ **Linoleic acid (18:2*n*-6):** Greatly increased (from 1.06% in raw to 43.50% in canned).<br>◦ **Long-chain n-3 PUFAs:** Concentrations decreased significantly (EPA: 4.86% to 0.84%; DHA: 29.46% to 4.34%).<br>◦ **SFA:** Decreased (25.18% to 13.81%).<br>◦ **MUFA:** Increased (9.71% to 21.42%).	
**Conclusion**	Heat processing in sealed cans had no significant effect on the fatty acid profile or lipid class composition; however, the use of vegetable oil as a packing medium has major effects on the product fatty acid profile, greatly increasing linoleic acid and decreasing the concentrations of long-chain polyunsaturated fatty acids	

<table>
<tr><td rowspan="5">Study Characteristics</td><td colspan="2">Description</td></tr>
<tr><td>Author, date</td><td>Aubourg et al. (1990)</td></tr>
<tr><td>Country</td><td>Spain</td></tr>
<tr><td>Publication type</td><td>Full text - Journal Article (J. Agric. Food Chem.)</td></tr>
<tr><td>Funding</td><td>Financial support from the Comisión Asesora de Investigación Científica y Técnica (Project PR 84-0043)</td></tr>
<tr><td></td><td>Conflict</td><td>None explicitly declared</td></tr>
<tr><td rowspan="8">Material & Methods</td><td>Aim of study</td><td>To investigate and compare lipids of the edible muscle and lipids from the fill oil to determine changes and interactions during canning and storage</td></tr>
<tr><td>Geographical area of catch</td><td>Atlantic Ocean (43° N and 27° W)</td></tr>
<tr><td>Fish type</td><td>Albacore tuna (Thunnus alalunga)</td></tr>
<tr><td>Filling medium</td><td>Soybean oil (SO)</td></tr>
<tr><td>Study design</td><td>Three different muscle parts (back, belly flap, ventral) were separated from steam-cooked tuna (102-103°C). Samples were canned in soybean oil, sterilized at 115°C for 60 min, and stored at room temperature</td></tr>
<tr><td>Duration of the study</td><td>Storage times of 5 days, 3 months, 7 months, and 11 months</td></tr>
<tr><td>FA analysis</td><td>Gas Chromatography (GC) using a 30-m flexible capillary column with SP-2330</td></tr>
<tr><td colspan="2"></td></tr>
<tr><td>Outcomes</td><td colspan="2">Canned Albacore Tuna in Soybean Oil:
◦ General: An interaction occurred between fish lipids and fill oil, manifested by an interchange of lipid molecules and fatty acids.
◦ Compared to initial (cooked) samples, canned tuna flesh:
▪ SFA: Decreased (e.g., from ~31–32% in cooked to ~22–24% in canned).
▪ MUFA: Decreased (e.g., from ~29–32% in cooked to ~25–26% in canned).
▪ PUFA: Significantly Increased (e.g., from ~28–33% in cooked to ~43–48% in canned) driven by the absorption of linoleic acid (18:2) from the soybean oil.
▪ EPA (20:5n-3): Decreased in percentage (e.g., from ~5.4% in cooked to ~2.7–4.2% in canned) due to dilution by the oil.
▪ DHA (22:6n-3): Decreased in percentage (e.g., from ~20% in cooked to ~12–18% in canned).
◦ Filling Oil: The oil showed significant increases in fatty acids characteristic of fish lipids (EPA and DHA) which were initially absent or low in the soybean oil</td></tr>
<tr><td>Conclusion</td><td colspan="2">The use of oil as a packing medium has significant effects on the lipid content and fatty acid profile of the final product, manifested by an interchange of lipid molecules where the flesh lipids show a steep increase in fatty acids abundant in the fill oil (18:2, 18:3), while the fill oil becomes enriched with characteristic fish fatty acids (22:6, 20:5); however, the total content of polyunsaturated fatty acids in the system remains unaltered by processing or storage</td></tr>
</table>

<table>
<tr><td rowspan="6">Study Characteristics</td><td colspan="2">Description</td></tr>
<tr><td>Author, date</td><td>García-Arias et al. (1994)</td></tr>
<tr><td>Country</td><td>Spain</td></tr>
<tr><td>Publication type</td><td>Full text - Journal Article (Journal of Food Composition and Analysis)</td></tr>
<tr><td>Funding</td><td>Spanish Comisión Asesora de Investigación Científica y Técnica (Project AL1 88-0255)</td></tr>
<tr><td>Conflict</td><td>None explicitly declared.</td></tr>
<tr><td rowspan="7">Material & Methods</td><td>Aim of study</td><td>To determine the quantitative and qualitative changes in white tuna fat throughout the different stages of processing (steaming, sterilization) and storage, and to evaluate modifications in the coating oil</td></tr>
<tr><td>Geographical area of catch</td><td>Atlantic Ocean (43° N and 27° W)</td></tr>
<tr><td>Fish type</td><td>Albacore tuna (Thunnus alalunga) (White tuna)</td></tr>
<tr><td>Filling medium</td><td>Soya bean oil (Soybean oil)</td></tr>
<tr><td>Study design</td><td>Thawed tuna were headed, eviscerated, and steamed (102–103∘C, 90 min). White meat was canned with soya bean oil and salt. Cans were sterilized at 115∘C for two different times: 55 minutes and 90 minutes.</td></tr>
<tr><td>Duration of the study</td><td>Storage times of 1 month (maturation), 1 year, and 3 years.</td></tr>
<tr><td>FA analysis</td><td>Gas Chromatography (GC) using a Hewlett Packard 5710 with a 6-foot stainless steel column packed with 10% Supelcoport 2330</td></tr>
<tr><td>Outcomes</td><td colspan="2">• Steaming: Did not produce remarkable changes in the percentage of fatty acid composition compared to raw tuna, though total fat content increased due to moisture loss.
• Canned Tuna in Soybean Oil (Compared to steamed samples):
◦ SFA: Palmitic acid (C16:0) tended to decrease.
◦ MUFA: Oleic acid (C18:1) increased significantly due to the filling oil.
◦ PUFA: Linoleic acid (C18:2) increased drastically (e.g., from ~1.3% in steamed to ~30–35% in canned). Linolenic acid (C18:3) also increased.
◦ EPA (20:5n-3): Decreased slightly after sterilization.
◦ DHA (22:6n-3): Decreased significantly (23–37% loss after sterilization) due to exchange with the oil.
◦ Ratios: The n-6/n-3 ratio increased significantly (7.7 to 11 times higher than in raw samples).
• Storage Effect: Changes observed after 1 year indicated that fatty acid exchanges maintained or increased, making the fish lipid profile more similar to the coating oil</td></tr>
<tr><td>Conclusion</td><td colspan="2">The fat composition of the canned tuna became similar to that of the soya bean oil used for coating, characterized by a decreased n-3 fatty acid content and an increased n-6 fatty acid content relative to raw or steamed samples; this effect was influenced by sterilization and was more evident after 1 and 3 years of storage</td></tr>
</table>

**Study Characteristics**	**Description**	
	**Author, date**	Medina et al. (1995)
	**Country**	Spain (Institute of Marine Research, CSIC, Vigo) and Italy (University of Naples)
	**Publication type**	Full text - Journal Article (*J. Sci. Food Agric.*)
	**Funding**	Research Project CEE UP.3-783 (of DG XIV) and Departamento de Postgrado (CSIC)
	**Conflict**	None explicitly declared.
**Material & Methods**	**Aim of study**	To investigate the effect of the filling medium (brine and soybean oil) on lipid modifications (specifically hydrolysis and lipid interchange) occurring during the industrial canning of tuna, utilizing Carbon-13 nuclear magnetic resonance (13C-NMR) spectroscopy
	**Geographical area of catch**	Atlantic Ocean (Atlantic Albacore)
	**Fish type**	Atlantic Albacore tuna (*Thunnus alalunga*)
	**Filling medium**	Brine (20 g/L NaCl), Soybean oil (with 2 g NaCl)
	**Study design**	Whole fish were steamed (102–103°C, 90 min) to a backbone temperature of 65°C. Cooked white muscle (90 g) was placed in cans with either brine or soybean oil. Cans were vacuum-sealed and sterilized at 110°C for 55 minutes.
	**Duration of the study**	Stored at room temperature for 3 months.
	**FA analysis**	Lipids were extracted using the Bligh and Dyer method. Quantitative analysis of lipid classes (FFA, TG, DG, PL) and fatty acid distribution/hydrolysis was performed using 13C-NMR spectroscopy (AC-270 Bruker spectrometer at 67.88 MHz)
**Outcomes**	**General Hydrolysis:** Sterilization caused an increase in FFA levels in both filling media. The extent and mechanism of lipolysis were found to be **independent** of the filling medium used,.<br>• **Hydrolysis Specificity:** There was a preferential hydrolysis of **PUFA** esterified in the *sn*-2 position of the glycerol moiety during sterilization.<br>• **Canned Tuna in Brine:**<br>◦ Showed a diminution in PUFA content related to preferential lipolysis compared to other fatty acids.<br>◦ Showed a high proportion of Phospholipids (PL), likely due to more effective extraction from the muscle in brine compared to oil, or a decrease in TG due to lipolysis.<br>• **Canned Tuna in Soybean Oil:**<br>◦ **Dilution Effect:** The natural lipids of the muscle were diluted by triacylglycerols from the filling medium.<br>◦ **Specific FA Changes:** This dilution resulted in an increase in the proportion of **OA** and **LA** (abundant in soybean oil) in the fish triacylglycerols,.<br>◦ **n-3 PUFA:** The amount of n-3 PUFA found in the esterified lipids was low due to dilution by the filling oil.	
**Conclusion**	The study concluded that sterilization induces preferential hydrolysis of PUFA esterified in the *sn*-2 position, a mechanism independent of the filling medium; however, the use of soybean oil as a filling medium leads to the absorption of vegetable triacylglycerols and a subsequent dilution of the natural marine lipids and n-3 PUFA in the fish muscle.	

<table>
<tr><td rowspan="6">Study Characteristics</td><td colspan="2">Description</td></tr>
<tr><td>Author, date</td><td>Ruiz-Roso et al. (1998)</td></tr>
<tr><td>Country</td><td>Spain</td></tr>
<tr><td>Publication type</td><td>Full text - Journal Article (J. Sci. Food Agric.)</td></tr>
<tr><td>Funding</td><td>Supported in part by Bernardo Alfageme SA</td></tr>
<tr><td>Conflict</td><td>None explicitly declared.</td></tr>
<tr><td rowspan="7">Material & Methods</td><td>Aim of study</td><td>To determine the effects of the canning and maturation processes on the fatty acid composition and on organoleptic characteristics (palatability) of sardines canned in olive oil</td></tr>
<tr><td>Geographical area of catch</td><td>Sada (Spain)</td></tr>
<tr><td>Fish type</td><td>Sardines (Sardina pilchardus)</td></tr>
<tr><td>Filling medium</td><td>Refined olive oil</td></tr>
<tr><td>Study design</td><td>Sardines were headed, eviscerated, and brined. Samples were taken at the raw stage (RS). Fish were steam-cooked (110°C, 30 min) and dried (PS). Refined olive oil was added, and cans were sterilized (115°C, 45 min). Samples were analyzed immediately after canning (CS), and after storage at room temperature</td></tr>
<tr><td>Duration of the study</td><td>Storage/maturation periods of 6 months (6MS), 12 months (12MS), and 5 years (5YS)</td></tr>
<tr><td>FA analysis</td><td>Lipids were extracted using a modified Bligh and Dyer method. Fatty acid methyl esters were analyzed by Gas Chromatography (Hewlett-Packard 5890-II) with a steel column packed with Supelcoport 2330</td></tr>
<tr><td>Outcomes</td><td colspan="2">Canning Process (Compared to Raw Sardines):
◦ SFA: Significant loss in fish fat. Palmitic acid (C16:0) decreased from ~273 g kg−1 in raw to ~169 g kg−1 in canned sardines,. Total SFA decreased from 415 g kg−1 (raw) to 243 g kg−1 (canned).
◦ MUFA: Significant rise due to olive oil absorption. Oleic acid (C18:1 n-9) increased from ~207 g kg−1 in raw to ~506 g kg−1 in canned sardines,. Total MUFA increased from 295 g kg−1 to 554 g kg−1.
◦ PUFA: n-6 PUFA increased (e.g., C18:2 n-6 from 21 to 37 g kg−1). n-3 PUFA decreased in concentration per kg of fat (e.g., EPA from 112 to 63 g kg−1; DHA from 54 to 45 g kg−1),.
◦ Filling Oil: The coating oil became enriched with SFA and n-3 PUFA transferred from the fish,.
• Storage/Maturation (up to 5 Years):
◦ Qualitative changes continued but were less significant than those during canning. SFA decreased slightly (to 203 g kg−1 at 5 years), and n-3 PUFA increased slightly (to 167 g kg−1 at 5 years) compared to just-canned samples,.
◦ Palatability: A sensory panel found that sardines stored for 6 months had significantly higher quality (appearance, texture, taste) than just-canned sardines. This quality was maintained for at least 5 years</td></tr>
<tr><td>Conclusion</td><td colspan="2">Canning sardines in olive oil produced slight quantitative modifications but very important qualitative changes in fatty acids, characterized by a significant loss of saturated fatty acids and an increase in mono-unsaturated fatty acids due to exchange with the coating oil; furthermore, the maturation process significantly improved palatability after 6 months, maintaining this quality for at least 5 years</td></tr>
</table>

<table>
<tr><td rowspan="6">Study Characteristics</td><td colspan="2">Description</td></tr>
<tr><td>Author, date</td><td>Rossi et al. (2001)</td></tr>
<tr><td>Country</td><td>Italy</td></tr>
<tr><td>Publication type</td><td>Ful text - Journal Article (Italian Journal of Food Science)</td></tr>
<tr><td>Funding</td><td>Not stated; however, preparation support was provided by Trinity Alimentari S.p.A.</td></tr>
<tr><td>Conflict</td><td>None explicitly declared</td></tr>
<tr><td rowspan="7">Material & Methods</td><td>Aim of study</td><td>To evaluate the lipid interchange between sardines and covering olive oil and to determine how this contact affects official methods for assessing oil genuineness</td></tr>
<tr><td>Geographical area of catch</td><td>Adriatic Sea</td></tr>
<tr><td>Fish type</td><td>Sardine (Sardina pilchardus)</td></tr>
<tr><td>Filling medium</td><td>Olive oil (blend of refined and virgin) and Water (used as a control to measure fat release without oil interference)</td></tr>
<tr><td>Study design</td><td>Sardines were thawed, headed, gutted, and steam-cooked on a grill for 10 minutes at atmospheric pressure. Pre-cooked fish (90g) were packed with 30g of filling medium, sealed, and sterilized at 121°C for 50 minutes.</td></tr>
<tr><td>Duration of the study</td><td>Storage for up to 4 months at room temperature, with analysis at 0, 30, 60, 90, and 120 days</td></tr>
<tr><td>FA analysis</td><td>Gas Chromatography (GC) using a 30-m Supelco SE 54 capillary column</td></tr>
<tr><td>Outcomes</td><td colspan="2">Canned Sardine Muscle (Lipid Interchange):
◦ Absorption: The fish muscle significantly adsorbed the filling oil during storage.
◦ MUFA: In the fish muscle lipids, oleic acid (C18:1 n-9) content rose from an initial level of ~7% in fresh sardines to approximately 30% after four months of storage in olive oil.
• Filling Oil Analysis (Enrichment from Fish):
◦ SFA: Increased in the filling oil (from 18.33% in the pure oil to ~22.15% after 120 days) due to transfer from the fish.
◦ MUFA: Decreased in the filling oil (from 65.88% initially to ~59.23% after 120 days) as it was diluted by fish lipids.
◦ PUFA: Increased in the filling oil (from 15.71% initially to ~18.72% after 120 days).
◦ EPA and DHA: These acids, initially absent in the olive oil, were detected immediately after sterilization. After 120 days of storage, the filling oil contained 1.57% EPA and 2.31% DHA</td></tr>
<tr><td>Conclusion</td><td colspan="2">A significant bidirectional lipid interchange occurs during the canning and storage of sardines in olive oil; the fish muscle adsorbs vegetable oil (increasing oleic acid and phytosterols), while the covering oil is enriched with fish lipids (increasing SFA and n-3 PUFAs like EPA and DHA), which can significantly alter the chemical indicators used to verify the genuineness of the olive oil</td></tr>
</table>

<table>
<tr><th></th><th colspan="2">Description</th></tr>
<tr><td rowspan="5">Study Characteristics</td><td>Author, date</td><td>Sadok, S., & Selmi, S. (2007)</td></tr>
<tr><td>Country</td><td>Tunisia</td></tr>
<tr><td>Publication type</td><td>Full text - Journal Article (Bull. Inst. Natn. Scien. Tech. Mer de Salammbô)</td></tr>
<tr><td>Funding</td><td>Not stated; however, support for sample supply was provided by the Sidi Daoud ABCO Fish Factory Corporation</td></tr>
<tr><td>Conflict</td><td>None explicitly declared</td></tr>
<tr><td rowspan="8">Material & Methods</td><td>Aim of study</td><td>To determine the effect of the canning process and the nature of the coating medium on the fatty acid profiles and lipid quality indicators of sardine flesh</td></tr>
<tr><td>Geographical area of catch</td><td>Sidi Daoud, Tunisia</td></tr>
<tr><td>Fish type</td><td>Sardinella aurita (Spanish sardine/Sardinella) and Sardina pilchardus (Sardine).</td></tr>
<tr><td>Filling medium</td><td>Olive oil (for S. aurita) and Tomato sauce (for S. pilchardus)</td></tr>
<tr><td>Study design</td><td>Fish were gutted, headed, and analyzed at three stages: raw, cooked (100°C for 30 min), and canned/sterilized (115°C for 40 min)</td></tr>
<tr><td>Duration of the study</td><td>Samples were analyzed immediately after processing and after 3 months of storage.</td></tr>
<tr><td>FA analysis</td><td>Gas Chromatography (GC) using an Agilent 6890N equipped with a polar INNOWAX 30 M silica capillary column</td></tr>
<tr><td colspan="2"></td></tr>
<tr><td>Outcomes</td><td colspan="2">Canned Spanish Sardine (S. aurita) in Olive Oil:
◦ SFA: Decreased from 53.57% to 46.40%.
◦ MUFA: Increased from 24.27% to 30.67%.
◦ PUFA: Remained relatively stable (17.54% to 19.09%).
◦ EPA: Decreased from 3.92% to 3.00%.
◦ DHA: Decreased from 6.09% to 5.12%.
• Canned Sardine (S. pilchardus) in Tomato Sauce:
◦ SFA: Decreased from 37.00% to 27.58%.
◦ MUFA: Increased from 11.34% to 23.05%.
◦ PUFA: Remained stable (44.03% to 41.92%; no significant difference).
◦ EPA: Decreased from 6.24% to 4.15%.
◦ DHA: Decreased from 33.61% to 24.79%.
• Lipid Interchange: Canned fish absorbed oleic (C18:1 n-9) and linoleic (C18:2 n-6) acids from the filling media, which diluted the original marine n-3 PUFAs</td></tr>
<tr><td>Conclusion</td><td colspan="2">The canning process and the choice of filling medium significantly alter the fatty acid profile of fish muscle through lipid interchange; nevertheless, the final canned products remain excellent nutritional sources of marine omega-3 and omega-6 fatty acids</td></tr>
</table>

	Description	
**Study Characteristics**	**Author, date**	Rasmussen et al. (2008)
	**Country**	USA
	**Publication type**	Full text - ournal Article (*Journal of Aquatic Food Product Technology*)
	**Funding**	Acknowledgment provided to Oregon Ocean Seafoods for use of their facility
	**Conflict**	None explicitly declared.
**Material & Methods**	**Aim of study**	To investigate the effects of canning and short-term storage on the fatty acid profile of once-cooked, raw-packed U.S. West Coast troll-caught albacore tuna
	**Geographical area of catch**	U.S. West Coast
	**Fish type**	Albacore tuna (*Thunnus alalunga*).
	**Filling medium**	Raw packed; no additional packing materials or precooking steps were used, in accordance with typical custom-canning procedures
	**Study design**	Dorsal loins from 13 fish were cut into cubes. Samples were analyzed at three stages: raw, immediately after canning (sterilized at 119°C for 72 min), and after 5 weeks of storage
	**Duration of the study**	5 weeks of storage at room temperature
	**FA analysis**	Gas Chromatography (GC) using a Shimadzu GC-2010 equipped with an Omegawax 250 capillary column
**Outcomes**	**Compared to initial (raw) samples, canned and stored tuna:**<br>◦ **SFA:** Remained stable (30.8% in raw vs. 30.3% at day 0 and 30.9% at week 5).<br>◦ **MUFA:** Remained stable (18.9% in raw vs. 19.3% at day 0 and 19.4% at week 5).<br>◦ **PUFA:** Remained stable (50.2% in raw vs. 50.4% at day 0 and 49.7% at week 5).<br>◦ **EPA:** Showed a **significant increase** immediately after canning (from 9.1% to 9.4%), but **returned to the original level** (9.1%) after 5 weeks of storage.<br>◦ **DHA:** Remained stable (33.8% in raw vs. 33.7% at day 0 and 33.2% at week 5).<br>• **Nutritional Density:** On a tissue weight basis, total n-3 PUFAs increased from 3.3 g/100 g in raw tuna to **4.0–4.1 g/100 g** in the canned product; this was due to moisture loss during canning, which concentrated the available lipids	
**Conclusion**	The study concluded that custom-canning of raw-packed, troll-caught albacore tuna does not lead to major alterations in the fatty acid profile or a reduction in nutritionally important n-3 PUFAs, as the absence of a filling medium prevents the lipid interchange typically seen in oil-packed canned fish	

<table>
<tr><td></td><td colspan="2">Description</td></tr>
<tr><td rowspan="5">Study Characteristics</td><td>Author, date</td><td>Selmi, S., Monser, L., & Sadok, S. (2008)</td></tr>
<tr><td>Country</td><td>Tunisia</td></tr>
<tr><td>Publication type</td><td>Full text - Journal Article (Journal of Food Processing and Preservation)</td></tr>
<tr><td>Funding</td><td>Not stated; acknowledgment given to Sidi Daoud Fish Factory Corporation for sample supply</td></tr>
<tr><td>Conflict</td><td>None explicitly declared</td></tr>
<tr><td rowspan="7">Material & Methods</td><td>Aim of study</td><td>To determine the chemical variation in fatty acids and nitrogenous compounds in two blue fish species canned under industrial conditions and stored for various periods</td></tr>
<tr><td>Geographical area of catch</td><td>Sidi Daoud, Tunisia</td></tr>
<tr><td>Fish type</td><td>Bluefin tuna (Thunnus thynnus) and Sardine (Sardina pilchardus)</td></tr>
<tr><td>Filling medium</td><td>Olive oil (for tuna) and Tomato sauce (for sardine)</td></tr>
<tr><td>Study design</td><td>Tuna were beheaded, gutted, filleted, and cooked for 1 hour at 100°C. Sardines were headed, gutted, and cooked in cans for 30 minutes at 100°C. Both were then sterilized at 115°C for 100 minutes</td></tr>
<tr><td>Duration of the study</td><td>Storage for 3 months and 6 months at ambient temperature</td></tr>
<tr><td>FA analysis</td><td>Gas Chromatography (GC) using an Agilent 6890N with a polar Innowax 30 M silica capillary column</td></tr>
<tr><td>Outcomes</td><td colspan="2">Compared to initial (fresh) samples:
• Canned Tuna in Olive Oil:
◦ SFA: Decreased from 39.22% to 25.22–26.56%.
◦ MUFA: Significantly increased from 27.33% to 49.21–49.24% due to olive oil absorption.
◦ PUFA: Slightly decreased from 21.46% to 19.01–20.19%.
◦ EPA: Decreased from 5.28% to 2.96–3.00%.
◦ DHA: Decreased from 11.96% to 5.16–5.85%.
• Canned Sardine in Tomato Sauce:
◦ SFA: Decreased from 37.00% to 26.42–27.00%.
◦ MUFA: Significantly increased from 11.34% to 23.73–23.84%.
◦ PUFA: Remained relatively stable (44.03% to 41.12–42.99%; no significant difference at 6 months).
◦ EPA: Decreased from 6.24% to 3.92–4.15%.
◦ DHA: Decreased from 33.61% to 23.69–26.31%.
• Lipid Interchange: Both species absorbed oleic (C18:1 n-9) and linoleic (C18:2 n-6) acids from the covering media during sterilization, enriching the fish flesh with these fatty acids while diluting original marine n-3 PUFAs.</td></tr>
<tr><td>Conclusion</td><td colspan="2">While the canning process and choice of filling medium slightly altered the fatty acid profiles, specifically increasing oleic and linoleic acid levels through oil absorption, the final canned tuna and sardine products remain excellent sources of both omega-3 and omega-6 fatty acids</td></tr>
</table>

	Description	
**Study Characteristics**	**Author, date**	Siriamornpun et al. (2008)
	**Country**	Thailand
	**Publication type**	Full text - Journal Article (*Journal of Food Lipids*)
	**Funding**	Mahasarakham University research grant
	**Conflict**	None explicitly declared
**Material & Methods**	**Aim of study**	To investigate the changes in content and composition of lipids and fatty acids of canned tuna in brine during a 12-month storage period
	**Geographical area of catch**	Not explicitly stated (samples supplied by a company in Bangkok, Thailand)
	**Fish type**	Tuna
	**Filling medium**	Brine (1% NaCl)
	**Study design**	Canned tuna samples were obtained from a single production batch and stored at room temperature. Samples were analyzed at baseline (0 months) and after 3, 6, 9, and 12 months
	**Duration of the study**	12 months
	**FA analysis**	Capillary gas chromatography (GC) using a 60-m fused silica bonded phase column DB-23
**Outcomes**	**Lipid Content:** Remained stable for the first 6 months but significantly **increased** after 9 months of storage.<br>• **Fatty Acid Profile Changes Over 12 Months:**<br>◦ **SFA:** Significantly **increased** after storage for 9 months. Specifically, palmitic (16:0) and stearic (18:0) acids increased after 6 months.<br>◦ **MUFA:** Showed a significant increase starting from 6 months of storage.<br>◦ **PUFA:** Stepwise significant **decrease** with increased storage time.<br>◦ **n-3 and n-6 PUFA:** Both concentrations significantly **decreased** over the storage period. n-3 PUFAs showed a greater overall loss (25%) compared to n-6 PUFAs (12%).<br>◦ **EPA:** Significantly **decreased** starting at 3 months of storage.<br>◦ **DHA:** Significantly **decreased** starting at 6 months of storage.<br>◦ **Ratio (n-3/n-6):** Significantly **decreased** from 3.8 at 0 months to 3.2 at 9 months.<br>• **Lipid Oxidation:** Measured by peroxide value (PV) and thiobarbituric acid (TBA) value, both showed gradual increases with storage time, though levels remained within safe consumption limits	
**Conclusion**	The study concluded that the concentrations of healthy polyunsaturated fatty acids, particularly omega-3s, significantly decline over time in brine-packed tuna; therefore, canned tuna in brine should not be stored for more than 6 months to ensure the stability and nutritional quality of its n-3 fatty acids	

	Description	
Study Characteristics	**Author, date**	Naseri, M., Rezaei, M., moieni, S., Hosseini, H., & Eskandari, S. (2011)
	**Country**	Iran
	**Publication type**	Full text - Journal Article (*International Journal of Food Science & Technology*)
	**Funding**	Not stated; however, technical assistance was acknowledged from several researchers
	**Conflict**	None explicitly declared
**Material & Methods**	**Aim of study**	To investigate the effect of four different filling media (sunflower oil, soybean oil, olive oil, and brine) on the oxidation and lipid quality of canned silver carp
	**Geographical area of catch**	Fish were transported from farms in Khuzestan, Iran
	**Fish type**	Silver carp (*Hypophthalmichthys molitrix*)
	**Filling medium**	Sunflower oil, Soybean oil, Olive oil, and Brine (20 g/L NaCl)
	**Study design**	Fish were beheaded, gutted, and filleted. Fillets were steamed (102–103°C) until a backbone temperature of 65–70°C was reached (48 min). Muscle portions (160 g) were placed in cans with 34 mL of filling medium and 2 g of NaCl, vacuum-sealed, and sterilized at 121°C for 65 min
	**Duration of the study**	4 months
	**FA analysis**	Gas Chromatography (GC) using a Shimadzu 17A equipped with a Carbowax 20M fused silica capillary column
**Outcomes**	**Compared to raw/precooked samples, canned silver carp flesh:**<br>◦ **SFA:** Decreased in all samples canned in vegetable oils (e.g., from **28.1%** raw to **19.8–22.5%** in oil); levels in brine-packed samples remained relatively stable (**25.1%**).<br>◦ **MUFA:** Significantly **increased** in the olive oil pack (to **54.5%**) due to oleic acid absorption, but decreased in soybean and sunflower oil packs.<br>◦ **PUFA:** Significantly **increased** in sunflower and soybean oil packs (rising from **15.6%** raw to **~33–35%**) due to the massive uptake of linoleic acid (18:2n-6) from the fill oils.<br>◦ **EPA (20:5n-3):** Decreased in percentage in all oil-canned flesh (down to **~1.7%**) due to dilution, but **increased** in brine-canned muscle (**2.75%**).<br>◦ **DHA (22:6n-3):** Remained relatively stable in the brine-canned muscle (**2.2%**) but showed a relative decrease in percentage in oil-canned samples due to the high volume of absorbed vegetable oils.<br>◦ *n*−3/*n*−6 **Ratio:** Decreased significantly in all oil-packed samples (most notably in sunflower oil, dropping from **1.42** to **0.24**) while remaining highest in brine-packed samples.<br>• **Filling Oil Analysis:** All vegetable oils showed a bidirectional exchange; the oils became enriched with marine fatty acids (EPA, DHA, and C14:0) that were initially absent or present in only trace amounts in the pure oils	
**Conclusion**	The study concluded that the fatty acid composition of canned silver carp is primarily determined by the filling medium used, with the fish muscle tending to resemble the fatty acid profile of the coating oil; furthermore, while oil canning dilutes the proportion of healthy marine *n*−3 PUFAs, brine-canning preserves these levels more effectively and results in the highest *n*−3/*n*−6 nutritional ratio	

<table>
<tr><td rowspan="6">Study Characteristics</td><td colspan="2">Description</td></tr>
<tr><td>Author, date</td><td>Naseri, M., & Rezai, M. (2012)</td></tr>
<tr><td>Country</td><td>Iran</td></tr>
<tr><td>Publication type</td><td>Full text - Journal Article (Journal of Aquatic Food Product Technology)</td></tr>
<tr><td>Funding</td><td>Not stated; however, technical assistance was acknowledged from several researchers</td></tr>
<tr><td>Conflict</td><td>None explicitly declared</td></tr>
<tr><td rowspan="7">Material & Methods</td><td>Aim of study</td><td>To determine the influence of canning and long-term (3-year) storage on the lipid and fatty acid composition of oil- and brine-canned sprat</td></tr>
<tr><td>Geographical area of catch</td><td>Caspian Sea</td></tr>
<tr><td>Fish type</td><td>Sprat (Clupeonella cultriventris)</td></tr>
<tr><td>Filling medium</td><td>Sunflower oil (+ salt) and Brine (aqueous NaCl)</td></tr>
<tr><td>Study design</td><td>Fish were beheaded, eviscerated, and steamed (102–103°C, 15 min). Portions of 160 g of cooked muscle were placed in 200 mL cans with the filling medium, sealed, and sterilized at 121°C for 65 min ($F0$=7 min)</td></tr>
<tr><td>Duration of the study</td><td>3 years of storage at room temperature, with analysis at 4 and 36 months</td></tr>
<tr><td>FA analysis</td><td>Gas Chromatography (GC) using a Shimadzu 17A equipped with a 50-m Carbowax 20M fused silica capillary column</td></tr>
<tr><td>Outcomes</td><td colspan="2">Canned Sprat in Sunflower Oil:
◦ SFA: Decreased from ~27.7% in cooked fish to 19.0% (at 4 months) and 18.9% (at 36 months).
◦ MUFA: Decreased significantly from ~30.7% in cooked fish to 27.7% (at 4 months) and 22.9% (at 36 months).
◦ PUFA: Significantly increased from ~33.7% in cooked fish to 50.0% (at 4 months) and 53.3% (at 36 months) due to absorption of the filling oil.
◦ n-6: Increased drastically (from ~3.7% raw to ~40.7% after 3 years) due to sunflower oil intake.
◦ n-3: Significantly decreased from ~30.0% raw to ~12.6% after 3 years.
◦ Ratio (n-3/n-6): Dropped significantly from 7.61 (raw) to 0.42 (after canning) and 0.31 after 3 years.
• Canned Sprat in Brine:
◦ Initial effects: Major fatty acid groups (SFA, MUFA, PUFA) did not significantly alter immediately after canning compared to cooked fillets.
◦ 3-Year storage: MUFA and -3 decreased significantly, while SFA increased to 30.76%.
◦ Ratio (n-3/n-6): Remained much higher than oil-packed samples but decreased slightly from 7.69 to 7.23 after storage.
• Lipid Damage: Free Fatty Acid (FFA) levels and tertiary oxidation products (fluorescence) increased significantly after sterilization and continued to rise over the 3-year period</td></tr>
<tr><td>Conclusion</td><td colspan="2">Canning sprat in sunflower oil significantly alters the fatty acid profile through bidirectional lipid interchange, drastically reducing the n-3/n-6 ratio; conversely, brine canning maintains a superior nutritional omega-3 balance, though both media experience increased lipid hydrolysis and oxidation during long-term (3-year) storage</td></tr>
</table>

	Description	
**Study Characteristics**	**Author, date**	Czerner, M., Agustinelli, S. P., Guccione, S., & Yeannes, M. I. (2015)
	**Country**	Argentina
	**Publication type**	Full text - Journal Article (*International Journal of Food Sciences and Nutrition*)
	**Funding**	Supported by grants from the Consejo Nacional de Investigaciones Científicas y Técnicas (CONICET) and the Universidad Nacional de Mar del Plata
	**Conflict**	One author was noted as an employee of OmegaSur S.A.
**Material & Methods**	**Aim of study**	To determine the effect of salting–ripening, marinating, and canning processes on the proximate chemical composition and fatty acid profile of anchovy, with an emphasis on long-chain polyunsaturated fatty acids (PUFAs)
	**Geographical area of catch**	Coastal region of Argentina, near the Mar del Plata Port
	**Fish type**	Anchovy (*Engraulis anchoita*)
	**Filling medium**	Soybean oil
	**Study design**	Beheaded and gutted fresh anchovies were steam pre-cooked at atmospheric pressure for 15 minutes. Fish were cooled, placed in cans with added salt (NaCl) and soybean oil, vacuum-sealed, and sterilized at 115°C for 90 minutes
	**Duration of the study**	Samples were analyzed at the raw stage, after pre-cooking, and as the final canned product
	**FA analysis**	Fatty acid methyl esters (FAMEs) were analyzed by Gas Chromatography (Shimadzu GC-17A) using an Omegawax 320 fused silica capillary column
**Outcomes**	**Compared to initial (fresh) samples:**<br>• **Lipid Content:** Decreased slightly after steam-cooking due to lipid release but **increased significantly** after sterilization (from 4.25% in raw to **6.23%** in canned) due to the absorption of the soybean oil.<br>• **Fatty Acid Profile Changes: MUFA:** Significantly decreased from ~36.80% in raw fish to **~20.56%** in canned fish, primarily due to the reduction in cetoleic acid (C22:1 n-11). **n-6 PUFA:** Significantly **increased** from ~8.69% in raw to **~28.45%** in canned muscle, specifically driven by the uptake of linoleic acid (C18:2 *n*-6) from the soybean oil. **n-3 PUFA (EPA and DHA):** The relative contents of essential EPA and DHA remained **comparable** between fresh and canned anchovy (EPA: 4.47% vs. 4.36%; DHA: 22.61% vs. 18.85%). **Ratio (n-3/n-6):** Considerably **reduced** from 3.75 in fresh fish to **0.94** in canned fish because of the absorption of the n-6-rich soybean oil. **Comparison to Other Processes:** The salting–ripening process led to the largest nutritional changes (a 70% reduction in total EPA and DHA), whereas canned and marinated anchovies maintained n-3 levels more similar to fresh samples	
**Conclusion**	While the canning process and the use of soybean oil significantly altered the fatty acid profile—most notably by increasing the n-6 content and reducing the n-3/*n*-6 ratio—canned anchovy remains a high-quality nutritional source, providing substantial amounts of essential EPA and DHA that satisfy a large portion of daily requirements	

<table>
<tr><td rowspan="6">Study Characteristics</td><th colspan="2">Description</th></tr>
<tr><td>Author, date</td><td>Mesías, M., Holgado, F., Sevenich, R., Briand, J. C., Márquez-Ruiz, G., & Morales, F. J. (2015)</td></tr>
<tr><td>Country</td><td>Spain and Germany</td></tr>
<tr><td>Publication type</td><td>Full text - Journal Article (Journal of Food and Nutrition Research)</td></tr>
<tr><td>Funding</td><td>Funded by the EU-FP7 PROMETHEUS project</td></tr>
<tr><td>Conflict</td><td>None declared</td></tr>
<tr><td rowspan="7">Material & Methods</td><td>Aim of study</td><td>To investigate the impact of conventional retort sterilization versus a novel alternative, high pressure thermal sterilization (HPTS), on the fatty acid composition of canned tuna and sardine</td></tr>
<tr><td>Geographical area of catch</td><td>Pacific Ocean (Tuna)
Mediterranean Sea (Sardine)</td></tr>
<tr><td>Fish type</td><td>Yellowfin tuna (Thunnus albacares) and Sardine (Sardina pilchardus)</td></tr>
<tr><td>Filling medium</td><td>Brine (for tuna), Sunflower oil (for tuna), and Olive oil (for sardine)</td></tr>
<tr><td>Study design</td><td>Retort (Conventional): Cans sterilized at 116°C for 60 minutes. HPTS (Alternative): Cans subjected to 600 MPa of pressure at 115°C for 28 minutes. The sterilization factor (F0) was held at 7 minutes for both treatments.</td></tr>
<tr><td>Duration of the study</td><td>The research involved four experimental batches collected between November 2011 and December 2013. Before processing, the fish were frozen for 10–12 weeks, and the study results focused on the immediate impact of sterilization treatments rather than long-term post-canning storage</td></tr>
<tr><td>FA analysis</td><td>Gas Chromatography (GC) using an HP Innowax capillary column</td></tr>
<tr><td>Outcomes</td><td colspan="2">Canned Tuna (Brine & Sunflower Oil):
◦ HPTS vs. Retort: The alternative HPTS treatment did not significantly affect the fatty acid profile compared to conventional retorting.
◦ The Polyene Index (PI), which measures lipid stability, remained stable across both treatments for tuna.
• Canned Sardine (Olive Oil):
◦ PUFA Loss: Unlike tuna, sardine samples treated with HPTS showed significantly lower total PUFA content compared to retorted samples.
◦ EPA & DHA: The sum of EPA and DHA in HPTS-treated sardines was nearly half of that found in retorted samples.
◦ Polyene Index (PI): The PI was significantly lower in HPTS samples (0.2) compared to retorted samples (0.5), indicating higher lipid alteration due to the combination of high pressure and heat.
• Mineral Impact: The researchers suggested that the higher mineral content (specifically iron) in sardines compared to tuna may have acted as a pro-oxidant, accelerating lipid degradation during the high-pressure process</td></tr>
<tr><td>Conclusion</td><td colspan="2">While High Pressure Thermal Sterilization (HPTS) is a promising alternative for tuna, it may negatively impact the nutritional quality of sardines by significantly reducing healthy omega-3 fatty acids; consequently, the study highlights that fish species with higher mineral content may be more susceptible to lipid oxidation under high-pressure conditions</td></tr>
</table>

	Description	
Study Characteristics	**Author, date**	Herawati, E. R. N., & Susanto, A. (2016)
	**Country**	Indonesia (Yogyakarta)
	**Publication type**	Full text - Journal Article (*Aquatic Procedia*)
	**Funding**	Supported by the Indonesian Institute of Sciences
	**Conflict**	None explicitly declared
**Material & Methods**	**Aim of study**	To investigate the effect of different brine concentrations on the nutrient content and fatty acid profile of canned catfish
	**Geographical area of catch**	Local district of Gunungkidul, Yogyakarta, Indonesia
	**Fish type**	Catfish (*Pangasius sutchi*)
	**Filling medium**	Brine at three different concentrations (1%, 2%, and 3% NaCl) and Water (0% NaCl) as a control
	**Study design**	Catfish portions (200g) were placed in sterilized cans with the filling medium, exhausted for 10 minutes, and sterilized at 121°C for 20 minutes
	**Duration of the study**	Samples were quarantined for 14 days after processing to ensure quality before analysis
	**FA analysis**	Gas Chromatography (GC)
**Outcomes**	**Nutrient Content Changes:**<br>◦ **Ash Content:** Increased significantly (by approximately **1.47%**) as the brine concentration increased, due to the high mineral content of the salt.<br>◦ **Fat Content:** Decreased by approximately **30%** with the addition of brine compared to the fresh fish.<br>◦ **Protein Content:** Remained stable; different brine concentrations had **no significant effect** on the protein levels of the catfish.<br>• **Fatty Acid Profile Changes:**<br>◦ **SFA:** The percentage of saturated fatty acids **increased** in the canned samples compared to fresh fish, becoming the dominant fatty acid group in the final product.<br>◦ **PUFA:** In fresh catfish, polyunsaturated fatty acids were the most abundant group; however, their relative percentage was influenced by the canning process and the medium used.<br>◦ **DHA:** Remained the dominant PUFA component in both fresh and canned samples (ranging from **0.64% in fresh** to **1.49% in water-packed** and **0.96% in 3% brine-packed** fish).<br>• **Medium Impact:** The study confirmed that the liquid medium (brine) transfers heat during the retort process and interacts with the fish muscle to alter the final fatty acid concentrations.	
**Conclusion**	The canning process and the use of brine significantly increase the mineral (ash) content and the proportion of saturated fatty acids in catfish, while simultaneously reducing the total fat content; despite these changes, canned catfish remains a source of beneficial fatty acids like linoleic acid, EPA, and DHA.	

	Description	
**Study Characteristics**	**Author, date**	Gómez-Limia, L., Cobas, N., Franco, I., & Martínez-Suárez, S. (2020)
	**Country**	Spain
	**Publication type**	Full text - Journal Article (*Food Research International*)
	**Funding**	Supported by the Xunta de Galicia
	**Conflict**	The authors declared no competing financial interests
**Material & Methods**	**Aim of study**	To evaluate the changes in fatty acid (FA) profiles produced at each step of the canning process and during storage, including the influence of different filling media and added spices
	**Geographical area of catch**	River Ulla, Galicia, NW Spain
	**Fish type**	European eel (*Anguilla anguilla*)
	**Filling medium**	Sunflower oil, Olive oil (a refined and virgin mixture), and **Spiced olive oil** (containing chili and four types of peppercorns)
	**Study design**	Eels were thawed in 12% brine, sliced, and fried at 190°C for 2 minutes (to eliminate water). The fried slices were packed in cans with hot filling media, vacuum-sealed, and sterilized at 118°C for 30 minutes (*F*0=11)
	**Duration of the study**	Samples were analyzed at the raw stage and after 2 and 12 months of storag
	**FA analysis**	Gas Chromatography (GC) using an Innowax column
**Outcomes**	**Compared to raw eel samples:**<br>◦ **SFA:** The total saturated fatty acid content declined from **32.0%** in raw fish to a range of **21.8–27.0%** post-canning due to the absorption of unsaturated filling oils.<br>◦ **MUFA:** Significantly **increased** in olive oil and spiced olive oil packs (reaching approximately **60%**) due to the massive absorption of oleic acid (C18:1n-9).<br>◦ **PUFA:** Content rose drastically in the sunflower oil pack (from **15.2%** raw to **~31.5%** canned) because of the uptake of linoleic acid (C18:2n-6).<br>◦ **EPA and DHA¯:** Both essential n-3 fatty acids decreased significantly during canning in all oil types; relative EPA fell from **3.66%** to **~2%** and DHA fell from **1.88%** to **~1%**.<br>◦ **Ratio (n-6/n-3):** Increased most significantly in sunflower oil-packed samples, rising from **0.41** in raw eels to **10.23** after 12 months of storage.<br>• **Storage and Media Effects:** Storage time increased oil absorption by the muscle, further aligning the fish's lipid profile with that of the filling oil. Notably, **spiced olive oil** resulted in lower losses of certain fatty acids (like C16:1 and C18:4n-3), suggesting that the spices exerted a **protective effect against lipid oxidation** during sterilization.	
**Conclusion**	The study concluded that while heat treatments and storage significantly alter the fatty acid profile of European eels through oil absorption and moisture-fat exchange, the final canned products remain high-quality nutritional sources; furthermore, the addition of spices like chili and pepper may help preserve healthy unsaturated fats by retarding oxidative deterioration	

	Description	
Study Characteristics	**Author, date**	Domiszewski, Z. (2021)
	**Country**	Poland
	**Publication type**	Full text - Journal Article (*Journal of Food Processing and Preservation*)
	**Funding**	Not stated
	**Conflict**	None declared
Material & Methods	**Aim of study**	To investigate the influence of sterilization on the behavior and true retention (TR) rate of EPA and DHA in the whole canned product (both solid and liquid parts)
	**Geographical area of catch**	FAO 27 (The Northeast Atlantic) for herring and mackerel, and FAO 27 IIId (Baltic Sea) for sprat.
	**Fish type**	Atlantic mackerel (*Scomber scombrus*), Atlantic herring (*Clupea harengus*), and Baltic sprat (*Sprattus sprattus*)
	**Filling medium**	Sunflower oil and Tomato sauce
	**Study design**	Frozen blocks of fish were thawed and immersed in a salt and acetic acid solution. Fish were packed in cans and steamed at 100°C for 15 minutes before adding the filling media. Cans were sterilized at 115°C for 50 minutes and analyzed after 14 days of maturation
	**Duration of the study**	3.5 months
	**FA analysis**	Gas Chromatography (GC) was used to measure EPA and DHA in percentages, absolute values (g/100g), and true retention rates
Outcomes	• **Whole Product Stability:** Total losses of EPA and DHA in the **whole canned product** (combining both the fish and the liquid medium) were relatively small and **did not exceed 7.5%**.<br>• **Retention in Fish Muscle:** After sterilization, the fish muscle retained between **64.8% and 71.1%** of the original EPA and DHA content found in the steamed raw material.<br>• **Regrouping (Lipid Interchange):** The "loss" of omega-3s from the fish muscle was not due to physical destruction but rather "**regrouping,**" where the fatty acids eluted from the fish into the surrounding oil or sauce.<br>• **Medium Impact:**<br>◦ In **oil-canned products**, the percentage share of EPA and DHA dropped significantly (by 20.2%–26.7%) due to "**dilution**" caused by the migration of vegetable oil into the fish meat.<br>◦ In **tomato sauce-canned products**, this dilution effect was generally **absent** because the sauce (an oil/water emulsion) did not diffuse into the fish as easily as pure oil.<br>• **Cooking Loss:** Sterilization caused a decrease in the weight of solid fish parts and the formation of a **water layer** in the liquid portion due to the thermal denaturation of proteins	
Conclusion	The study concluded that the canning process is not as destructive to EPA and DHA as previously believed, as the majority of these essential fatty acids remain within the can; however, because they migrate into the filling media, it is recommended that the liquid part of the can be consumed rather than discarded to achieve the full nutritional benefit	

<table>
<tr><td rowspan="6">Study Characteristics</td><td colspan="2">Description</td></tr>
<tr><td>Author, date</td><td>Domiszewski, Z., & Mierzejewska, S. (2021)</td></tr>
<tr><td>Country</td><td>Poland</td></tr>
<tr><td>Publication type</td><td>Full text- Journal Article (International Journal of Food Science)</td></tr>
<tr><td>Funding</td><td>Not stated</td></tr>
<tr><td>Conflict</td><td>The authors declared no conflict of interest</td></tr>
<tr><td rowspan="7">Material & Methods</td><td>Aim of study</td><td>To investigate the influence of the technological process (specifically the "double dose" of heat from smoking and sterilization) on the true retention (TR) rate of EPA and DHA, lipid oxidation, and physical properties of canned sprat</td></tr>
<tr><td>Geographical area of catch</td><td>Baltic Sea</td></tr>
<tr><td>Fish type</td><td>Baltic sprat (Sprattus sprattus)</td></tr>
<tr><td>Filling medium</td><td>Sunflower oil</td></tr>
<tr><td>Study design</td><td>The study compared fresh fish and frozen fish (stored at -22°C for 4 months). Both groups were brined (20% NaCl), smoked (drying and natural smoke), beheaded, and packed in oval cans with sunflower oil. Cans were sterilized at 115°C for 50 minutes</td></tr>
<tr><td>Duration of the study</td><td>4 months</td></tr>
<tr><td>FA analysis</td><td>Gas Chromatography coupled with Mass Spectrometry (GC-MS)</td></tr>
<tr><td>Outcomes</td><td colspan="2">• Physical Changes (Cooking Loss): Sterilization caused a decrease in the weight of solid fish parts (by ~8%) and a corresponding increase in the liquid parts (~15%) as water was expelled from the muscle. Frozen fish had a higher "water layer" in the final can than fresh fish, indicating lower water-binding capacity.
• Lipid Content: Fat content in the wet tissue increased significantly after sterilization (by ~19% compared to smoked fish) due to both dehydration and the absorption of the sunflower oil.
• Fatty Acid Profile Changes:
◦ The percentage share of EPA and DHA in fish lipids decreased by 22.9%–30.3% after sterilization.
◦ This was caused by "dilution" as sunflower oil migrated into the meat—proven by a 5-fold increase in linoleic acid (C18:2n-6) in the fish.
• True Retention (TR): After sterilization, the fish muscle retained 70%–77% of its original EPA and DHA content. The "missing" portion was not destroyed by heat but had "regrouped" (migrated) into the filling oil.
• Fresh vs. Frozen Impact: Canned products made from fresh fish retained ~10% more EPA and DHA in the muscle than those made from frozen fish.
• Oxidation Levels: While primary and secondary oxidation products (PV and p-AsV) increased due to heat and salt, they remained well within the limits for "good quality" lipids (PV < 10 mEqO2/kg; p-AsV < 20)</td></tr>
<tr><td>Conclusion</td><td colspan="2">The study concluded that the canning process is not as destructive to omega-3 fatty acids as often assumed; instead, these healthy fats are mainly redistributed into the packing oil. Furthermore, the quality of the raw material is critical, as fresh fish preserves higher levels of essential fatty acids through the canning process compared to frozen fish</td></tr>
</table>

	Description	
**Study Characteristics**	**Author, date**	Bouriga, N., Rjiba-Bahri, W., Mili, S., Massoudi, S., Quignard, J. P., & Trabelsi, M. (2022)
	**Country**	**Tunisia** (Tunis and Bizerte) and France (Montpellier)
	**Publication type**	Full text - Journal Article (*Journal of Food Science and Technology*)
	**Funding**	Not applicable
	**Conflict**	The authors declared no conflict of interest
**Material & Methods**	**Aim of study**	To analyze an innovative method for canning sardine and compare the effects of grape seed oil (GSO) and olive oil (OO) on nutritional characteristics, fatty acid composition, and lipid stability
	**Geographical area of catch**	Fresh sardines were purchased from a local fish landing center in Bizerte, northeastern Tunisia
	**Fish type**	Sardine (*Sardina pilchardus*)
	**Filling medium**	Refined olive oil and cold-pressed grape seed oil (extracted in the laboratory from the Carignan grape cultivar)
	**Study design**	Sardines were gutted, headed, and packed into tin-free-steel cans. Sterilization was conducted at 121.5°C for 90 minutes
	**Duration of the study**	Samples were analyzed fresh and after 90 days (3 months) of storage
	**FA analysis**	Fatty acid methyl esters (FAME) were identified using Gas Chromatography (Agilent 6890N) with an Innowax capillary column
**Outcomes**	• **Nutrient Content Changes:**<br>◦ Canning significantly **reduced moisture content** (from 75% raw to 40–42% canned) while **increasing protein, ash, and lipid levels**.<br>◦ Sardines canned in GSO reached a lipid level of **12%**, compared to 6.4% in fresh fish, due to oil absorption and dehydration.<br>• **Fatty Acid Profile Changes:**<br>◦ The coating oil significantly altered the fish's profile; GSO-packed sardines became rich in **linoleic acid (C18:2n-6)**, which rose from 1.46% in fresh fish to **20.86%** after storage.<br>◦ Total **saturated fatty acids (SFA) decreased** from 40.86% in fresh fish to 33.22% (GSO) and 25.52% (OO).<br>◦ While relative EPA and DHA percentages decreased due to "dilution" from the vegetable oils, **DHA remained the most abundant unsaturated fatty acid** in all samples.	
**Conclusion**	The study concludes that using grape seed oil for canning is a highly beneficial innovation; it not only preserves the essential omega-3s of the sardine but also enriches the product with polyphenols and linoleic acid, creating a high-quality functional food that may help prevent cardiovascular disease and diabetes	

<table>
<tr><th></th><th colspan="2">Description</th></tr>
<tr><td rowspan="5">Study Characteristics</td><td>Author, date</td><td>Prego, R., Trigo, M., Martínez, B., & Aubourg, S. P. (2022)</td></tr>
<tr><td>Country</td><td>Spain</td></tr>
<tr><td>Publication type</td><td>Full text - Journal Article (Marine Drugs)</td></tr>
<tr><td>Funding</td><td>The research received no external funding.</td></tr>
<tr><td>Conflict</td><td>The authors declared no conflict of interest.</td></tr>
<tr><td rowspan="7">Material & Methods</td><td>Aim of study</td><td>To investigate the impact of prior frozen storage, different packing media, and the canning procedure on the fatty acid (FA) composition of Atlantic mackerel.</td></tr>
<tr><td>Geographical area of catch</td><td>Fresh mackerel were obtained at Vigo harbour in Northwestern Spain.</td></tr>
<tr><td>Fish type</td><td>Atlantic mackerel (Scomber scombrus).</td></tr>
<tr><td>Filling medium</td><td>Water, Brine (2% NaCl), Sunflower oil, Refined olive oil, and Extra virgin olive oil (EVOO).</td></tr>
<tr><td>Study design</td><td>◦ Prior Storage: One group was canned fresh (0-canned), while another was kept in frozen storage at -18°C for 6 months before canning (6-canned).
◦ Sterilization: Cans were sterilized in a steam retort at 115°C for 45 minutes ($F0$=7 min).</td></tr>
<tr><td>Duration of the study</td><td>Following sterilization, all cans were stored at room temperature for 3 months to allow for maturation before analysis.</td></tr>
<tr><td>FA analysis</td><td>Gas-liquid chromatography (GLC)</td></tr>
<tr><td>Outcomes</td><td colspan="2">• Canning Process Impact: The canning procedure itself led to a significant decrease in saturated fatty acids (STFA) and an increase in total polyunsaturated fatty acids (PUFA) and total omega-3 FA values.
• Packing Medium Effects:
◦ Sunflower Oil: Led to the highest PUFA levels but the lowest omega-3/omega-6 ratios due to the high presence of linoleic acid ($C18:2n{-}6$) in the oil.
◦ Olive Oils: While the olive oils were rich in oleic acid ($C18:1n{-}9$), this did not lead to a remarkable increase of that specific fatty acid in the canned fish muscle.
◦ Aqueous vs. Oily: Fish canned in water or brine showed higher lipid values than those in oily media; the authors suggested that oily packing media partially extracts the lipid fraction from the fish muscle.
• Frozen Storage Impact: Prior frozen storage for 6 months did not have a substantial effect on the final fatty acid group contents (STFA, MUFA, PUFA, or total omega-3) or the nutritional ratios.
• Nutritional Quality: All canned samples remained highly valuable for human health, maintaining $n{-}3/n{-}6$ ratios between 8.2 and 10.9 and high absolute levels of EPA and DHA</td></tr>
<tr><td>Conclusion</td><td colspan="2">The study concluded that while the choice of packing medium (particularly sunflower oil) can shift the fatty acid profile of the fish muscle, the canning of Atlantic mackerel results in a highly nutritional product regardless of whether the raw material was fresh or previously frozen for six months.</td></tr>
</table>

1. Hale, M.B.; Brown, T. Fatty acids and lipid classes of three underutilized species and changes due to canning. *Mar Fish Rev* **1983**, *45*, 45-48.
2. Aubourg, S.P.; Sotelo, C.G.; Gallardo, J.M. Changes in Flesh Lipids and Fill Oils of Albacore (Thunnus-Alalunga) during Canning and Storage. *J Agr Food Chem* **1990**, *38*, 809-812, doi:10.1021/jf00093a047.
3. García-Arias, M.T.; Sánchez-Muniz, F.J.; Castrillón, A.M.; Pilar Navarro, M. White Tuna Canning, Total Fat, and Fatty Acid Changes during Processing and Storage. *J Food Compos Anal* **1994**, *7*, 119-130, doi:10.1006/jfca.1994.1011.
4. Medina, I.; Sacchi, R.; Aubourg, S.P. A 13C‐NMR study of lipid alterations during fish canning: Effect of filling medium. *J Sci Food Agr* **1995**, *69*, 445-450, doi:10.1002/jsfa.2740690407.
5. Ruiz-Roso, B.; Cuesta, I.; Perez, M.; Borrego, E.; Pérez-Olleros, L.; Varela, G. Lipid composition and palatability of canned sardines.: Influence of the canning process and storage in olive oil for five years. *J Sci Food Agr* **1998**, *77*, 244-250, doi:10.1002/(Sici)1097-0010(199806)77:2<244::Aid-Jsfa34>3.0.Co;2-B.
6. Rossi, M.; Colonello, A.; Alamprese, C. Influence of lipid interchange between canned sardines and covering olive oil on some oil genuineness features. *Ital J Food Sci* **2001**, *13*, 159-171.
7. Selmi, S.; Sadok, S. Change in lipids quality and fatty acids profile of two small pelagic fish: Sardinella aurita and Sardina pilchardus during canning process in olive oil and tomato sauce respectively. *INSTM Bull Mar Freshw Sci* **2007**, *34*, 91-97, doi:10.71754/instm.bulletin.v34.669
8. Rasmussen, R.S.; Morrissey, M.T.; Roblero, J. Fatty Acid Composition of US West Coast Albacore Tuna (Thunnus alalunga) and the Effects of Canning and Short-Term Storage. *J Aquat Food Prod T* **2008**, *17*, 441-458, doi:10.1080/10498850802369211.
9. Selmi, S.; Monser, L.; Sadok, S. The influence of local canning process and storage on pelagic fish from Tunisia: Fatty acid profiles and quality indicators. *J Food Process Pres* **2008**, *32*, 443-457, doi:10.1111/j.1745-4549.2008.00189.x.
10. Siriamornpun, S.; Yang, L.F.; Kubola, J.; Li, D. Changes of omega-3 fatty acid content and lipid composition in canned tuna during 12-month storage. *Journal of Food Lipids* **2008**, *15*, 164-175, doi:10.1111/j.1745 4522.2007.00109.x.
11. Naseri, M.; Rezaei, M.; Moieni, S.; Hosseini, H.; Eskandari, S. Effects of different filling media on the oxidation and lipid quality of canned silver carp (Hypophthalmichthys molitrix). *Int J Food Sci Tech* **2011**, *46*, 1149-1156, doi:10.1111/j.1365-2621.2011.02608.x.
12. Naseri, M.; Rezaei, M. Lipid Changes During Long-Term Storage of Canned Sprat. *J Aquat Food Prod T* **2012**, *21*, 48-58, doi:10.1080/10498850.2011.582232.
13. Czerner, M.; Agustinelli, S.P.; Guccione, S.; Yeannes, M.I. Effect of different preservation processes on chemical composition and fatty acid profile of anchovy (Engraulis anchoita). *Int J Food Sci Nutr* **2015**, *66*, 887-894, doi:10.3109/09637486.2015.1110687.
14. Mesías, M.; Holgado, F.; Sevenich, R.; Briand, J.C.; Márquez Ruiz, G.; Morales, F.J. Fatty acids profile in canned tuna and sardine after retort sterilization and high pressure thermal sterilization treatment. *J Food Nutr Res* **2015**, *54*, 171–178
15. Herawati, E.R.N.; Angwar; Susanto, A.; Kurniadi. Effect of Brine Concentration on the Nutrient Content and Fatty Acid Profile of Canned Catfish [Pangasius Sutchi (Fowler, 1937)]. *Aquat Pr* **2016**, *7*, 85-91, doi:10.1016/j.aqpro.2016.07.011.
16. Gomez-Limia, L.; Cobas, N.; Franco, I.; Martinez-Suarez, S. Fatty acid profiles and lipid quality indices in canned European eels: Effects of processing steps, filling medium and storage. *Food Res Int* **2020**, *136*, 109601, doi:10.1016/j.foodres.2020.109601
17. Domiszewski, Z. Effect of sterilization on true retention rate of eicosapentaenoic and docosahexaenoic acid content in mackerel (Scomber scombrus), herring (Clupea harengus), and sprat (Sprattus sprattus) canned products. *J Food Process Preserv* **2021**, *45*, e15461, doi:10.1111/jfpp.15461.
18. Domiszewski, Z.; Mierzejewska, S. Effect of Technological Process on True Retention Rate of Eicosapentaenoic and Docosahexaenoic Acids, Lipid Oxidation and Physical Properties of Canned Smoked Sprat (Sprattus sprattus). *Int J Food Sci* **2021**, *2021*, 5539376, doi:10.1155/2021/5539376.
19. Bouriga, N.; Rjiba Bahri, W.; Mili, S.; Massoudi, S.; Quignard, J.P.; Trabelsi, M. Variations in nutritional quality and fatty acids composition of sardine (Sardina pilchardus) during canning process in grape seed and olive oils. *J Food Sci Technol* **2022**, *59*, 4844-4852, doi:10.1007/s13197-022-05572-4.
20. Prego, R.; Trigo, M.; Martinez, B.; Aubourg, S.P. Effect of Previous Frozen Storage, Canning Process and Packing Medium on the Fatty Acid Composition of Canned Mackerel. *Mar Drugs* **2022**, *20*, doi:10.3390/md20110666.
