# Supplementary material for: Systematic Review of Fatty Acid Composition and the Influence of Coating Media on Fatty Acid Profiles in Canned Fish
Source: Mar Drugs. 2026 Jun 10;24(6):204. doi: 10.3390/md24060204 (PMC13302670; doi:10.3390/md24060204)
Supplement: Supplementary file 1 [file marinedrugs-24-00204-s001.zip › SupplFiles/Supplementary_file_S1_Search strategies.pdf]

Supplementary file S1: Search strategies

MEDLINE (Ovid) Search Strategy

```
1.  exp Canning/
2.  exp Food Preservation/
3.  exp Seafood/
4.  (canned fish or canned seafood or preserved fish or fish canning
        or canned tuna or canned sardine or canned mackerel
        or canned salmon).tw.
5.  (canning adj3 fish).tw.
6.  (retort* adj3 fish).tw.
7.  or/1-6
8.  exp Fatty Acids/
9.  exp Fatty Acids, Unsaturated/
10. exp Fatty Acids, Omega-3/
11. exp Eicosapentaenoic Acid/
12. exp Docosahexaenoic Acids/
13. (fatty acid* or PUFA* or polyunsaturated fatty acid* or n-3
        or omega-3 or EPA or DHA).tw.
14. or/8-13
15. (filling medium or covering oil or liquid medium or brine
        or tomato sauce or sunflower oil or olive oil or soybean
oil).tw.
16. (packing medium or fill oil or coating medium or coating media).tw.
17. or/15-16
18. 7 and 14 and 17

Exp = MeSH term or descriptor
```

Scopus (www.scopus.com – Advanced search)

```
TITLE-ABS-KEY(
  ("canned fish" OR "canned seafood" OR "fish canning" OR "canned tuna"
   OR "canned sardine" OR "canned mackerel" OR "canned salmon"
   OR "preserved fish")
  AND
  ("fatty acid" OR "fatty acid composition" OR "polyunsaturated fatty
acid"
   OR "essential fatty acids" OR PUFA OR EPA OR DHA OR omega-3)
  AND
  ("filling medium" OR "coating medium" OR "covering oil" OR "coating
media"
   OR "liquid medium" OR brine OR "tomato sauce" OR "sunflower oil"
   OR "olive oil" OR "soybean oil")
)
```

Web of Science — Basic search (Topic):

```
TS = (
  ("canned fish" OR "canned seafood" OR "preserved fish" OR "fish
canning"
   OR "canned tuna" OR "canned sardine" OR "canned mackerel" OR "canned
salmon")
  AND
  ("fatty acid" OR "fatty acid composition" OR "polyunsaturated fatty
acid"
   OR "essential fatty acids" OR PUFA OR EPA OR DHA OR omega-3)
  AND
  ("filling medium" OR "coating media" OR "covering oil" OR "coating
medium"
   OR brine OR "tomato sauce" OR "sunflower oil" OR "olive oil" OR
"soybean oil")
)
```

Wiley Online Library — Advanced search:

```
("canned fish" OR "canned seafood" OR "fish canning" OR "canned tuna"
 OR "canned sardine" OR "canned mackerel" OR "canned salmon")
AND
("fatty acid" OR PUFA OR "polyunsaturated fatty acid*" OR EPA OR DHA OR
omega-3)
AND
("filling medium" OR "coating media" OR "covering oil" OR brine
 OR "tomato sauce" OR "sunflower oil" OR "olive oil" OR "soybean oil")
```

Cochrane Central Register of Controlled Trials (CENTRAL)

```
#1  MeSH descriptor: [Canning] explode all trees
#2  MeSH descriptor: [Seafood] explode all trees
#3  (canned fish OR canned seafood OR fish canning OR canned tuna
        OR canned sardine OR canned mackerel OR canned salmon):TI,AB,KY
#4  #1 OR #2 OR #3
#5  MeSH descriptor: [Fatty Acids] explode all trees
#6  MeSH descriptor: [Fatty Acids, Omega-3] explode all trees
#7  (PUFA* OR EPA OR DHA OR "fatty acid*" OR omega-3*):TI,AB,KY
#8  #5 OR #6 OR #7
#9  (filling medium OR covering oil OR brine OR tomato sauce
        OR sunflower oil OR olive oil OR soybean oil):TI,AB,KY
#10 #4 AND #8 AND #9
```

Cochrane Library (Reviews / Trials)

```
(canned fish OR canned seafood OR fish canning OR canned salmon)
AND (fatty acid OR PUFA OR EPA OR DHA OR omega-3)
AND (filling medium OR coating medium OR covering oil OR brine
     OR tomato sauce OR sunflower oil OR olive oil OR soybean oil)
```

Google Scholar (Grey Literature) — First 500 results screened

```
"canned fish" AND "fatty acids"
AND ("filling medium" OR "coating media" OR "covering oil"
     OR brine OR "tomato sauce")
```

Global Index Medicus (WHO)

```
("canned fish" OR "canned seafood" OR "preserved fish")
AND ("fatty acid" OR PUFA OR EPA OR DHA OR omega-3)
AND ("filling medium" OR "covering oil" OR brine
     OR "tomato sauce" OR "sunflower oil" OR "olive oil" OR "soybean
oil")
```
